# The cutting-edge advancements in biomaterials under the guidance of intelligence and bionics

**DOI:** 10.1093/rb/rbag110

**Published:** 2026-06-05

**Authors:** Yanjiao Teng, Jianing Yang, Tianmeng Li, Zihe Yin, Qi An, Ziqi Shi, Dagong Jia, Qi Lv, Seeram Ramakrishna, Jie Shi

**Affiliations:** Institution of Disaster and Emergency Medicine, Tianjin University, Tianjin 300072, China; Department of Mechanical Engineering, Tsinghua University, Beijing 100084, China; Institution of Disaster and Emergency Medicine, Tianjin University, Tianjin 300072, China; Key Laboratory of Medical Rescue Key Technology and Equipment, Ministry of Emergency Management, Tianjin 300072, China; Institution of Disaster and Emergency Medicine, Tianjin University, Tianjin 300072, China; Key Laboratory of Medical Rescue Key Technology and Equipment, Ministry of Emergency Management, Tianjin 300072, China; The Third Central Clinical College of Tianjin Medical University, Tianjin 300070, China; Institution of Disaster and Emergency Medicine, Tianjin University, Tianjin 300072, China; Institution of Disaster and Emergency Medicine, Tianjin University, Tianjin 300072, China; Key Laboratory of Medical Rescue Key Technology and Equipment, Ministry of Emergency Management, Tianjin 300072, China; Institution of Disaster and Emergency Medicine, Tianjin University, Tianjin 300072, China; Key Laboratory of Medical Rescue Key Technology and Equipment, Ministry of Emergency Management, Tianjin 300072, China; Institution of Disaster and Emergency Medicine, Tianjin University, Tianjin 300072, China; Key Laboratory of Medical Rescue Key Technology and Equipment, Ministry of Emergency Management, Tianjin 300072, China; Department of Mechanical Engineering, Tsinghua University, Beijing 100084, China; Institution of Disaster and Emergency Medicine, Tianjin University, Tianjin 300072, China; Key Laboratory of Medical Rescue Key Technology and Equipment, Ministry of Emergency Management, Tianjin 300072, China

**Keywords:** biomaterial, intelligence, bionics, regenerative medicine, clinical application

## Abstract

The fusion of biomaterials with intelligent technologies and bionics represents a significant transformation in modern medicine, particularly in therapeutic applications and regenerative medicine. The development history of biomaterials has gone through four distinct stages: from inert biological materials and materials with biological activity and biodegradability to stimulus-responsive biomaterials and finally to the current integration of intelligent bionic materials. Due to clinical needs and the impetus of precision medicine, this development process has also been accelerated by advanced technologies, including artificial intelligence (AI), 4D bioprinting and intracellular monitoring. Here, we introduced two major categories of advanced biomaterials: intelligent responsive materials and biomimetic functionalized materials. Key manufacturing and characterization platforms were also explored, including 4D printing technology for dynamic molding, design methods based on AI for rapid screening and *in vivo* monitoring techniques for real-time feedback. The cutting-edge clinical applications were introduced, including precise drug delivery and personalized medicine approaches within regenerative medicine. Although these technologies have made significant progress, there are still major challenges in laboratory-to-clinical applications, especially involving complex material-biological interfaces, long-term stability and the need for evolving regulatory frameworks. Future development depends on interdisciplinary collaboration, ultimately achieving a true symbiotic relationship between biomaterials and living systems.

## Graphical abstract

**Graphical Abstract:**
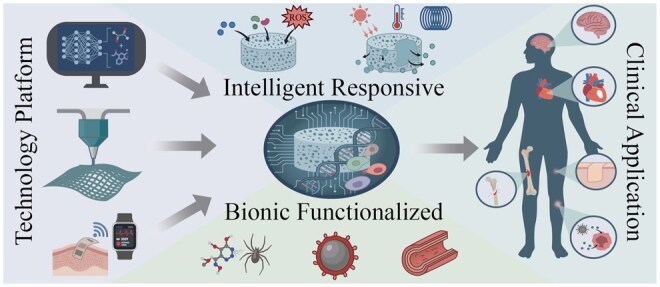

## Introduction

The cross-fusion of biomaterials with modern medicine and materials science significantly contributes to the forefront fields of tissue and organ repair and assisted disease treatment [1]. With the rapid development of various technologies, the design concept of biomaterials has also undergone changes and developments at different times. The flourishing development of intelligent and biomimetic materials is driven by urgent global social challenges and clinical needs. On the one hand, the global population is aging, and the incidence of osteoporosis fractures, articular cartilage degradation, cardiovascular diseases, neurodegenerative diseases and other diseases is soaring [2, 3]. The tissue regeneration ability of elderly patients is also lower, making the functional limitations of traditional materials more apparent, and there is an urgent need for emerging biomaterials to be developed [4]. On the other hand, the rise of precision medicine requires materials to be tailored to individual differences of patients for personalized design.

Patients with different causes and different disease subtypes have significant differences in their functional requirements for the implanted scaffold materials [5]. The same material is difficult to simultaneously meet the treatment needs of all patients [6, 7]. Osteoporosis and diabetes are two representative causes that lead to difficulties in bone defect healing [8–10]. For patients with osteoporosis, the core issue lies in the imbalance of bone metabolism throughout the body and the reduction in bone density [11, 12]. The core challenge of implanting a scaffold is how to achieve stable mechanical support in an environment with insufficient bone mass and promote local bone regeneration [13]. In contrast, the impaired bone healing in diabetic patients is the result of multiple factors working together. For example, high blood sugar inhibits the migration and proliferation of bone marrow mesenchymal stem cells, causing them to differentiate into adipocytes rather than osteoblasts [14]; the high sugar environment disrupts immune homeostasis, and the imbalance of macrophage polarization leads to persistent chronic inflammatory responses, further hindering the bone repair process [15], etc. Bone repair scaffold materials for diabetic patients cannot merely play the role of mechanical support or passive signal conduction but need to have the ability to actively sense and regulate the pathological microenvironment [16]. Precision medicine achieves this by deeply analyzing the individual differences in these pathological environments, thereby guiding the development of intelligent materials with targeted functions [17].

**Figure 1:**
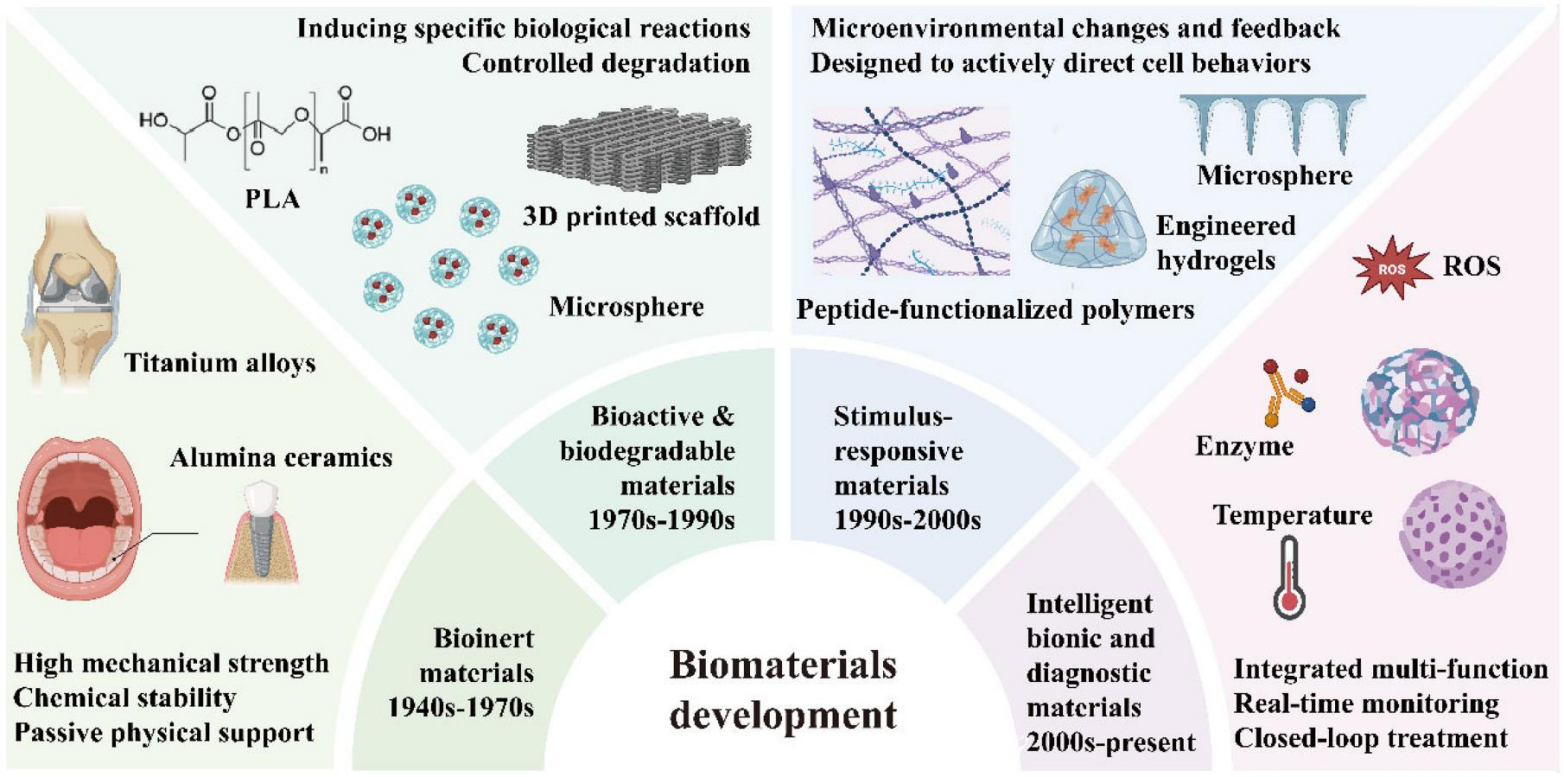
Schematic diagram of the four development stages of biomaterials. The diagram categorizes the evolution into four distinct levels based on their core characteristics: bioinert materials, bioactive & biodegradable materials, stimulus-responsive materials and intelligent materials.

Looking back at the development history of biomaterials, it can be basically divided into four landmark stages (Figure 1). The first generation is a biologically inert material with good mechanical strength and chemical stability, mainly used to replace damaged tissue’s physical functions and reduce host immune rejection reactions [18]. Typical representatives include alumina ceramics, titanium alloys, medical-grade polyethylene, silicone rubber, etc. [1, 18, 19]. However, they are essentially considered foreign objects, isolated from surrounding tissues by forming a fibrous encapsulation, lacking biological features for interaction with the biological environment, and can only provide passive physical support, making it difficult to achieve true tissue repair and regeneration [18, 20]. Therefore, the second generation possesses biological activity and degradability, inducing specific biological reactions through controlled chemical degradation or surface ion exchange. Polylactic acid, polyglycolic acid, and their copolymers are typical representatives [21]. By using electrospinning and 3D printing technology, these degradable polymers can be processed into nanofiber scaffolds and simulate the morphology of natural collagen fibers, significantly enhancing cell adhesion, proliferation and differentiation, and inducing tissue regeneration [22]. Afterwards, these materials hydrolyze and break chains and degrade, thus freeing up space for new tissue growth [23]. However, the human physiological environment is dynamic, and in complex pathological environments, a single degradation function is no longer sufficient to meet treatment needs. This has given rise to the third generation of stimulus-responsive biomaterials. They can perceive changes in the microenvironment, like pH value, temperature, reactive oxygen species (ROS), enzyme concentration, etc., and make corresponding physical and chemical feedback [24]. With the help of advanced technologies such as coaxial spinning or emulsion electrospinning, multilevel fibers can be constructed to precisely control the release of growth factors (GFs), gene carriers or small molecule drugs, greatly improving the accuracy of treatment [25]. For example, a decrease in pH in the tumor microenvironment or inflammation site can trigger swelling or degradation of drug-loaded nanofibers, achieving targeted drug release [26]. Introducing disulfide bonds into the polymer skeleton can respond to high concentrations of glutathione or ROS levels in cells and achieve on-demand degradation [27, 28].

Currently, biomaterials are developing towards the fourth generation, which is the integration of intelligent biomimetic and diagnostic materials, further promoting the realization of personalized precision medicine [1, 29–31]. For instance, MXene is a new type of two-dimensional transition metal carbides, nitrides or carbon nitrides. The progress in MXene-based stimulus-responsive materials and their evolution towards autonomous intelligent materials represents a specific research direction at the material system level of the ‘transition from response to autonomy’ [32]. Based on environmental responsiveness, they integrate multiple functions into the system, which can integrate external control functions such as light, electricity and magnetism. They can also be combined with electronic components to monitor biological signals in real time and achieve closed-loop treatment [33–35]. For instance, researchers use conductive materials like polyaniline and polypyrrole to prepare flexible neural interfaces and cardiac patches, regulating cell behavior via electrical signal conduction and promoting synchronous repair of nerve or myocardial tissue [36]. These materials have transformed from passive to active based on the previous three generations, capable of sensing pathological signals and dynamically regulating their own functions, making them an active therapeutic system for precise intervention in biological processes.

Lately, the maturity of advanced manufacturing technologies such as AI, 4D bioprinting and intracellular monitoring, it has provided great assistance for the design of complex biomaterials. AI and machine learning algorithms assist researchers in predicting the structure-activity relationship of polymers, thereby quickly screening formulations with optimal mechanical properties and biological functions, greatly shortening the research and development cycle [37, 38]. Introducing the concept of 4D bioprinting, researchers have utilized shape memory polymers (SMPs) to prepare dynamically evolving nanofiber scaffolds. After implantation, the stent can undergo pre-programmed shape changes under the stimulation of body temperature or specific ions, achieving self-unfolding or a perfect fit to irregular defect areas [39]. Synthetic biology technology assists in the precise customization of recombinant proteins with specific sequences and functions and prepares hybrid materials that combine natural protein biological activity and synthetic polymer mechanical stability through electrospinning by blending with synthetic polymers [40, 41].

The majority of the literature covered in this review is from the past five years. Most of the existing similar reviews focused on a single material, a single technology or a single clinical application. Our review focuses on integrating intelligent responsive materials with biomimetic functionalized materials, forming an integrated framework. At the same time, we provide detailed discussions on 4D printing and precise forming technology, an AI-powered material design platform, and in-body monitoring technology. We systematically analyzed their applications in the preparation and characterization of intelligent biomimetic biological materials. Secondly, our introduction covers six major areas of progress and clinical applications: skin wound repair, bone and cartilage regeneration, cardiovascular diseases, nerve injury repair, precise drug delivery and cancer treatment, as well as personalized implants and organ chips. Finally, we conduct a detailed examination of the existing obstacles and prospects of intelligent biomimetic biological materials. This review aims to build a bridge between advanced material design, intelligent manufacturing and clinical applications, providing a roadmap for future research on intelligent biomimetic biological materials.

## Intelligent and biomimetic biomaterials

### Intelligent responsive biomaterials

**Figure 2:**
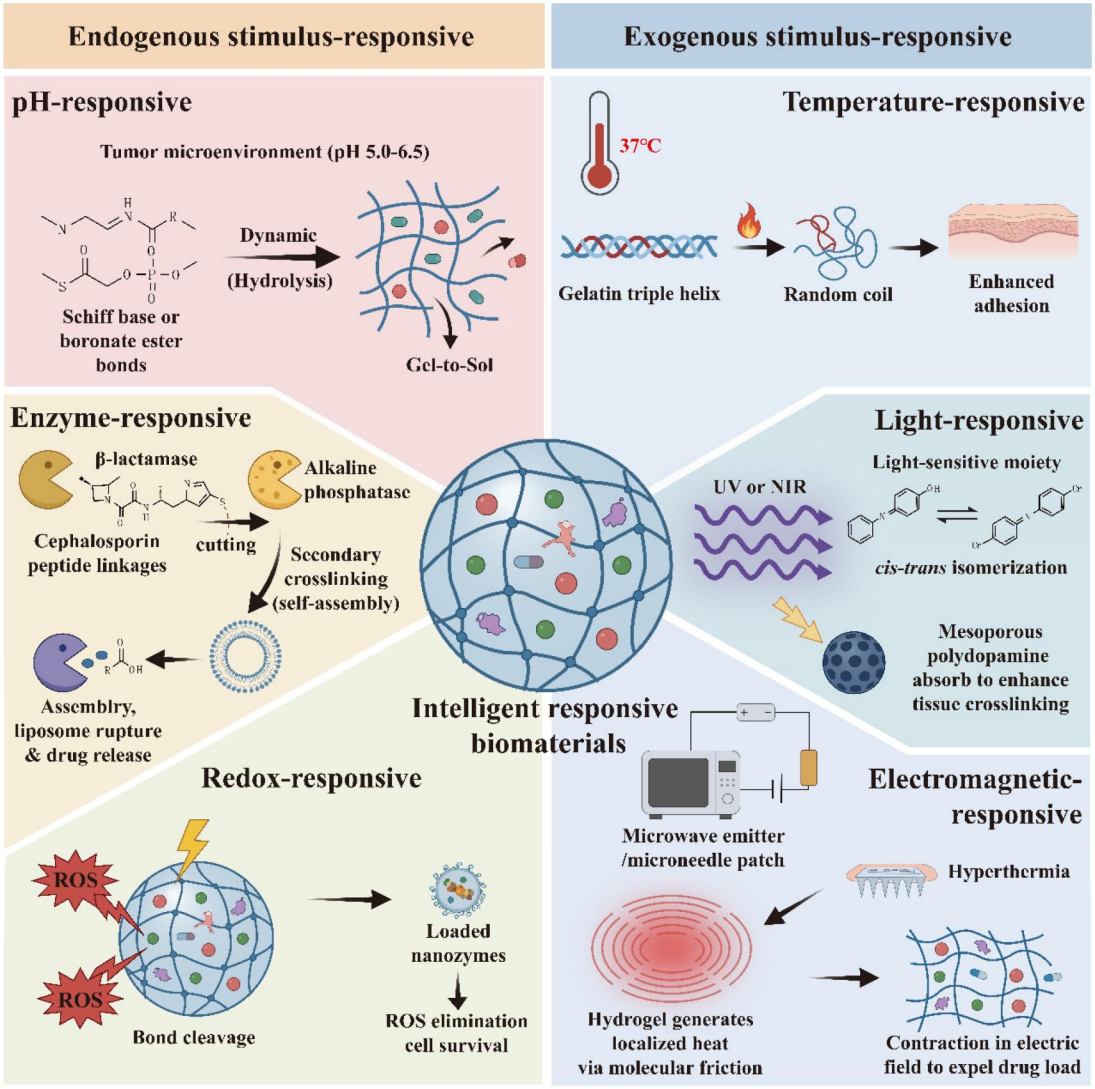
Schematic illustration of the classification of stimulus-responsive strategies for intelligent biomaterials. Endogenous stimulus-responsive biomaterials include pH-responsive, enzyme-responsive and redox-responsive biomaterials. Exogenous stimulus-responsive biomaterials include temperature-responsive, light-responsive and electromagnetic-responsive biomaterials.

In contrast to conventional biomaterials, which primarily serve as passive carriers, intelligent biomaterials represent a paradigm shift toward active regulation, functioning as adaptive materials that sense and respond to environmental cues by dynamically modulating their properties [42]. Intelligent biomaterials can be systematically classified based on the source and nature of the stimulus signals they respond to: endogenous stimulus-responsive materials (e.g. pH-responsive, enzyme-responsive and redox-responsive) respond to signals unique to the pathological or physiological microenvironment within the organism. Exogenous stimulus-responsive materials (e.g. temperature-responsive, optically-responsive and thermally-responsive) respond to physical stimuli applied by external devices, enabling noninvasive, spatiotemporal control [23] (Figure 2). The core design principle of intelligent responsive materials is the stimulus-response functionality, which is usually employed as an intelligent switch or actuator to control drug release, dynamic scaffold deformation or on-demand therapeutic intervention [42].

#### pH-responsive materials

Due to the different pH levels and gradients of each tissue, employing pH-responsive materials enables modulation at the target tissue [43]. pH-responsive materials have great promise for site-specific drug delivery, particularly in oncology. The application of pH-responsive strategies for tumor targeting has been extensively studied. Owing to the distinctive acidic nature of the tumor microenvironment compared to normal tissues, pH-responsive materials commonly incorporate polyanions or polycations, which undergo protonation or ionization upon pH changes or integrate pH-sensitive linkages into the therapeutic materials [44, 45]. For instance, the protonation of tertiary amine groups in the poly(dimethylaminoethyl methacrylate) backbone upon acidification in endosomes induces endosomal disruption, facilitating the release of the immunotherapeutic agent into the cytoplasm [46].

Given the dynamic pH variations associated with normal physiological processes and disease states, pH-responsive systems have proven to be indispensable tools in modern biomedical applications [47]. pH-responsive hydrogels can undergo volume phase changes (swelling/shrinking) or gel-sol transitions according to environmental pH variations. For swelling-shrinking type hydrogels, their response behavior stems from the protonation or deprotonation process of ionizable groups in the polymer network as pH changes [48]. Such hydrogels can be further classified into three types: anionic, cationic and amphoteric. For example, anionic hydrogels have acidic groups such as carboxyl (-COOH) or sulfonic acid (-SO_3_H) on their polymer chains, and typical polymers include polyacrylic acid (PAA) and polymethacrylic acid (PMAA) [49]. At low pH, the carboxyl groups protonate to the nonionized -COOH state, and the polymer chain adopts a compact coiled structure, causing the hydrogel to shrink. As pH increases, the carboxyl groups deprotonate to the negatively charged -COO^-^, and the polymer backbone unfolds owing to electrostatic repulsion, leading to significant swelling of the hydrogel [50].

For sol-gel type hydrogels, their response behavior is usually attributed to the reversible creation and dissociation of dynamic covalent linkages [51]. These hydrogels undergo reversible transitions between gel state and solution state (sol state) in response to pH changes, involving three types of dynamic covalent bonds: borate ester bond, hydrazone bond and imine bond (Schiff base) [52–54].

The Schiff base is one of the most representative pH-sensitive dynamic covalent linkages, created through the condensation of an aldehyde group with a primary amine. This reaction occurs under alkaline to neutral conditions, and in acidic conditions, the Schiff base hydrolyzes, leading to the dissociation of the hydrogel network [55]. For instance, the *in situ* formed hydrogel prepared by the imine formation between the amine groups of chitosan and the terminal formyl groups of polyether polyurethane is stable at pH 7.4. However, it gradually dissolves in an acidic environment with a pH of 5.0 due to the Schiff base hydrolysis [56].

**Figure 3:**
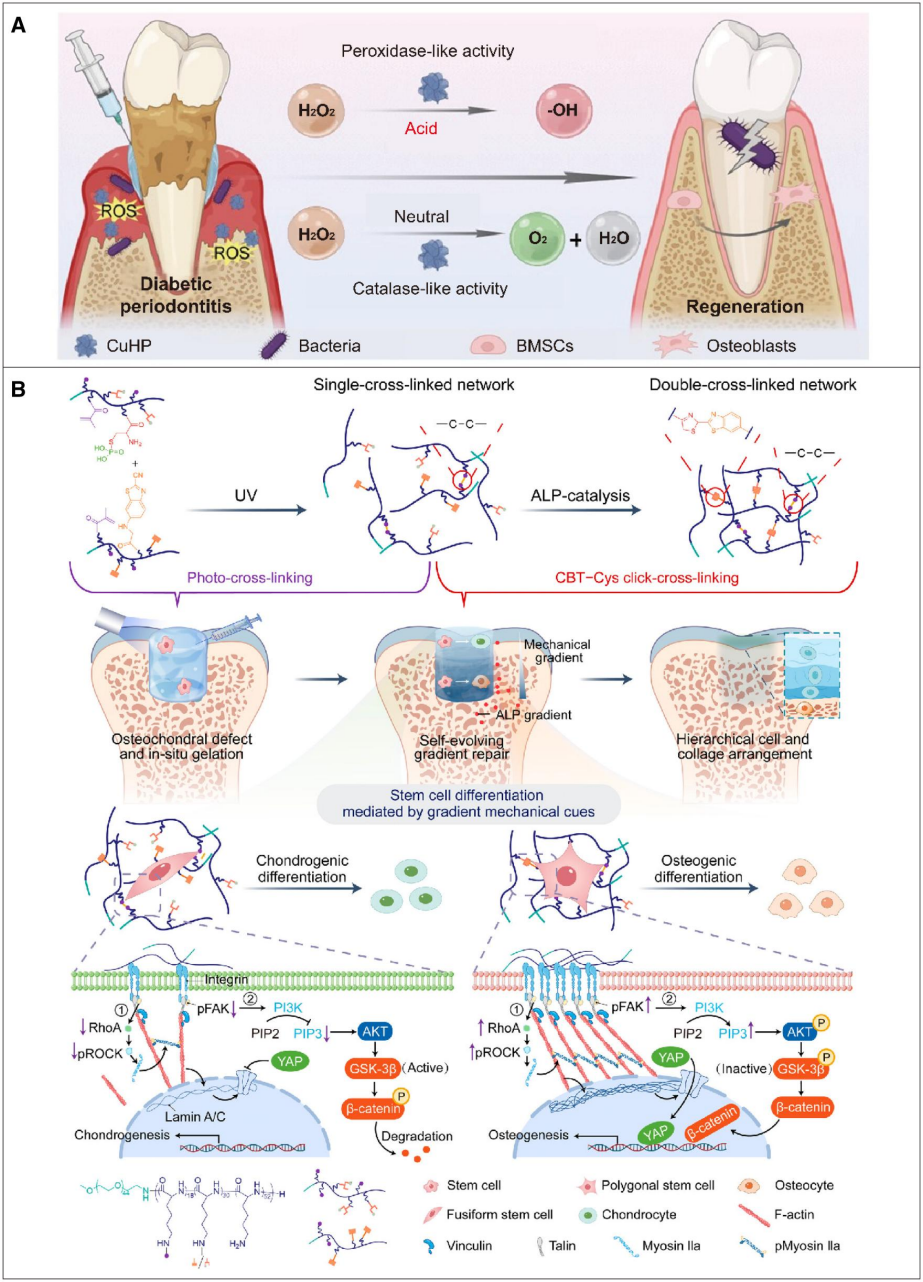
Intelligent materials with pH response and enzyme response. (**A**) pH-responsive composite hydrogel for the treatment of diabetic periodontitis. Reproduced with permission from Ref. [57]. Copyright © 2025 Oxford University Press. (**B**) The design of enzyme-responsive self-evolving mechanical gradient hydrogels and their mechanism of action in bone chondral regeneration. Reproduced with permission from Ref. [60]. Copyright © 2025 American Chemical Society.

Lu *et al.* [57] synthesized a copper hydrogen phosphate (CuHP) composite hydrogel. In an acidic environment under an infected state, this hydrogel mainly exhibits peroxidase (POD)-like activity, catalyzing the generation of ROS to efficiently kill periodontal pathogenic bacteria. In a neutral environment after the inflammation is controlled, its function transforms into catalase (CAT)-like activity, effectively removing excessive ROS in the tissue, reducing oxidative stress damage to bone marrow-derived mesenchymal stem cells, and synergistically promoting bone regeneration by combining with the continuous release of copper ions. In a rat model of diabetic periodontitis, the number of bacteria decreased significantly by approximately 75.7%. This hydrogel markedly suppressed bacterial proliferation and facilitated alveolar bone repair (Figure 3A).

#### Enzyme-responsive materials

Enzymes, as biological catalysts, exhibit high specificity and selectivity towards their substrates [58]. Due to the significant role enzymes play in various biological processes, abnormal enzymes related to diseases can also become a target in medicine, making enzyme reactions promising candidates for biomedical applications [59]. Recently, a study was reported that, for the first time, combined adaptive hydrogels with an alkaline phosphatase activity gradient. Zhuang *et al.* [60] developed a self-evolving hydrogel (SE gel) with enzyme-responsive spatiotemporal mechanical cues. It forms a secondary crosslinking network through the reaction between 2-cyanobenzothiazole, catalyzed by alkaline phosphatase and cysteine. This enzymatic crosslinking increases the network density and supplements the primary photo-crosslinked structure, thereby increasing the storage modulus by four times. Furthermore, in the 3D reconstruction images of the joints in the SE gel group, dense new bone formation was observed. Quantitative analysis showed that the bone volume/total volume in the SE group was significantly increased by 1.8 times compared to the static network group (SN). This hydrogel can provide dynamic and gradient mechanical signals during bone chondrocyte tissue repair to precisely regulate cell fate (Figure 3B). By using enzymes as the triggering agents for mechanical reactions, many potential applications have been opened up in regenerative medicine, diagnosis and drug delivery. The issue of antibiotic resistance in wound infections has become a global public health crisis [61]. Bacteria that produce β-lactamase are one of the main pathogenic bacteria causing clinical failure in anti-infection treatment [62]. Abbas *et al.* [62] designed and constructed a β-lactamase-responsive hydrogel, using cephalosporin derivatives as a shearable cross-linking agent and encapsulating liposomes loaded with ciprofloxacin. This intelligent hydrogel, with its triple capabilities of precise recognition, active capture and efficient removal, precisely captures drug-resistant bacteria, providing a new strategy for combating superbug infections.

The complex pathological microenvironment often requires the collaborative action of multiple enzymes. Ma *et al.* [63] constructed a zinc-coordinated tri-enzyme nanogel system (Zn@nGSC) by encapsulating glucose oxidase (GOx), superoxide dismutase (SOD) and CAT inside the imidazole-functionalized polymer nanogel scaffold, thereby imitating the natural enzyme assembly. Zn@nGSC demonstrated therapeutic effects on hyperglycemia and promotion of regeneration in a mouse model of wound healing after pancreatic resection by restoring redox balance and metabolic homeostasis. The Zn^2+^ ions can exert antibacterial effects, further promoting angiogenesis, collagen deposition and immune regulation.

#### Redox-responsive materials

Redox-responsive materials can respond to changes in the redox signals within the body and make corresponding functional adjustments. The design and application of these materials aim to simulate the redox regulation mechanisms within living organisms [64]. By interacting with ROS, reducing agents or other redox-related substances in the environment, they can achieve functions such as drug release, tissue repair and immune regulation [65].

Redox-responsive materials can be categorized into oxidizing-responsive materials and reducing-responsive materials [66]. In particular, ROS-responsive materials represent a crucial and cutting-edge direction in the field of oxidizing-responsive materials. Responsive biomaterials based on ROS can be activated by the ROS within the damaged tissue microenvironment, releasing their payloads or exerting therapeutic effects, thereby regulating the elevated ROS concentrations, reducing oxidative stress and promoting tissue regeneration [65, 67]. Wang *et al.* [68] used polyvinyl alcohol (PVA) and 2-amino-4-hydroxy-6-methylpyrimidine modified gelatin (Gel-UPy) to load membrane-coated nanozymes (MBC) and prepared a ROS-responsive nanozyme delivery system, PG@MBC. The borate ester bonds in the pathological microenvironment of the system respond and break, enabling the on-demand release of the nanozyme. PG@MBC not only can eliminate intracellular ROS (maintaining a clearance rate of >80% within 28 days) and inhibit ferroptosis of nuclear cells but also can effectively downregulate the expression of IL6. The study verified that PG@MBC could promote the dual repair of intervertebral disc architecture and function in a rat model of intervertebral disc degeneration and maintain mechanical properties for 12 weeks.

#### Temperature-responsive material

**Figure 4:**
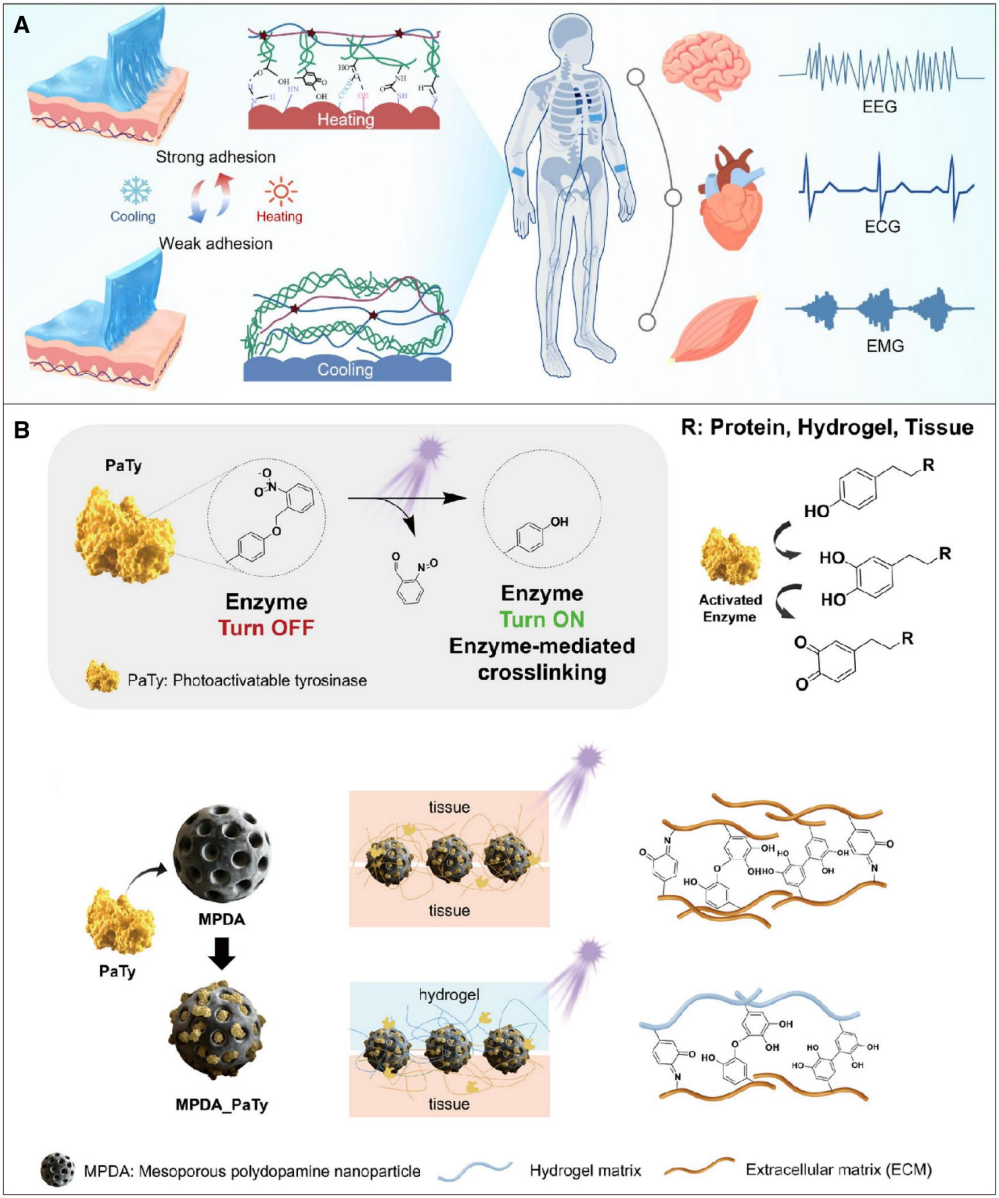
Intelligent materials related to temperature response and light response. (**A**) A conductive hydrogel that is temperature-controlled and has adjustable adhesiveness. Reproduced with permission from Ref. [72]. Copyright 2024 © Elsevier. (**B**) Schematic illustration of photoactivatable tyrosinase (PaTy) activation and schematic illustration of PaTy-loaded mesoporous polydopamine nanoparticle and the crosslinking of tissue-tissue and tissue-hydrogel interfaces by light-activated MPDA_PaTy. Reproduced with permission from Ref. [79]. Copyright 2025 © Wiley-VCH GmbH.

The core of temperature-responsive biomaterials lies in the capacity to undergo significant and reversible physical property changes in response to specific temperature variations [69]. These materials are typically composed of polymers with specific phase transition temperatures, and their macroscopic properties (such as solubility, volume and mechanical strength) can be precisely controlled within the physiological temperature range [70, 71]. The existing flexible bio-electrodes have defects such as weak adhesion and unstable signal collection. The temperature-triggered adhesive ionic conductive hydrogel was fabricated by Li *et al.* [72] using a combination of polyacrylamide (PAAM), gelatin and sodium alginate (SA). Through the temperature-sensitive gelatin component, it can quickly respond to the physiological temperature. Upon contact with human skin, the hydrogel is exposed to physiological temperature, which induces the denaturation of gelatin’s triple helix structure, leading to increased interfacial contact and improved adhesion to the skin; after cooling, the triple helix structure re-enters the coil state, reducing the adhesion to the skin, thus achieving painless removal. Based on the five-cycle adhesion tests of the hydrogel at different temperatures, the hydrogel maintains a relatively high average adhesion strength of 9 kPa at 37°C, while the adhesion strength is lower at 25°C. The hydrogel exhibits stable cyclic adhesion triggered by temperature (Figure 4A).

Temperature-responsive materials also offer new ideas for wound treatment. Huang *et al.* [73] developed a temperature-triggered self-contracting dressing composed of nanofibers and hydrogel. The electrospun nanofibers of poly(lactic acid-co-trimethylene carbonate) possess a glass transition temperature of approximately 37°C, aligning with human physiological temperature. This makes them act like a precise temperature sensor, capable of accurately sensing the temperature changes at the wound site. This research innovatively combines mechanical and biochemical signals, offering a novel strategy for diabetic wound treatment.

#### Light-responsive materials

Light-responsive biomaterials are the most spatially and temporally resolved and controllable type within the intelligent material system. The core of these materials lies in using specific wavelengths of light as a noninvasive and remotely and precisely controllable external ‘switch’ to trigger the predetermined physical or chemical changes in the materials [74, 75]. Light responsiveness originates from photosensitive groups within the material, which undergo molecular configuration or electronic state changes upon absorbing photons [76]. For example, in materials based on optical isomerization, photosensitive groups (such as azobenzene and lactone) undergo reversible cis-trans isomerization or ring-opening/ring-closing reactions after absorbing specific wavelengths of light, resulting in significant changes in the molecular geometry, dipole moment and hydrophilicity/hydrophobicity [77]. Light-responsive biomaterials also include materials based on photobleaching and those based on photothermal effects. They find extensive utility in biomedical domains like light-controlled drug delivery and precision chemotherapy, as well as photodynamic/photothermal synergistic therapy [78].

Light-response materials also hold significant promise for tissue engineering applications in wound healing. Ko *et al.* [79] proposed a spatiotemporal light-responsive and cooperative system: photoactivatable tyrosinase-loaded mesoporous polydopamine nanoparticles (MPDA_PaTy). Under ultraviolet (UV) irradiation, MPDA_PaTy can precisely regulate the bonding strength, and its bonding strength can be increased by up to 3.7 times. In a murine model of dorsal excisional wounds, the MPDA_PaTy cohort demonstrated a significantly accelerated wound closure rate in comparison to the other experimental groups. MPDA_PaTy, as the photo-activated adhesion platform, holds great potential in an extensive range of tissue engineering and regenerative medicine (Figure 4B).

#### Electromagnetic-response materials

The defining feature of electro-responsive materials is their capacity for reversible physicochemical transformations upon electric field application [80]. The defining feature of magnetic-responsive materials is their responsiveness to external magnetic fields. The core characteristic is attributed to the capability of remotely and accurately manipulating their location, shape, heat generation or mechanical behavior through non-contact magnetic fields, which offers unique advantages in the field of biomedicine [81].

Electromagnetic functional materials exhibit significant interaction with electromagnetic waves and are capable of modulating the frequency and intensity of the waves. This interaction forms the basis of their diverse functions [82, 83]. The electromagnetic response of these materials mainly depends on four core factors: charge transport, dielectric polarization, magnetic response and phenomena such as reflection/scattering [84]. Electromagnetic-responsive materials can also suppress the progression of primary and metastatic tumors. The microwave-sensitive immunological hydrogel strategy for tumor ablation therapy was developed by Sun *et al.* [85]. They combined local hyperthermia with immunotherapy by incorporating the immunoadjuvant R837 to synthesize alginate (ALG)-Ca^2+^ hydrogels loaded with R837. The (ALG)-Ca^2+^ hydrogel, through its inherent abundant ions and porous network structure, enhances the molecular friction and the spatial density of water molecules under microwave irradiation, thereby achieving efficient microwave thermal conversion. It is a typical microwave-sensitive biological material. Under microwave irradiation, the hydrogels generate heat locally, leading to thermal ablation of tumors with negligible harm to surrounding healthy tissues.

**Figure 5:**
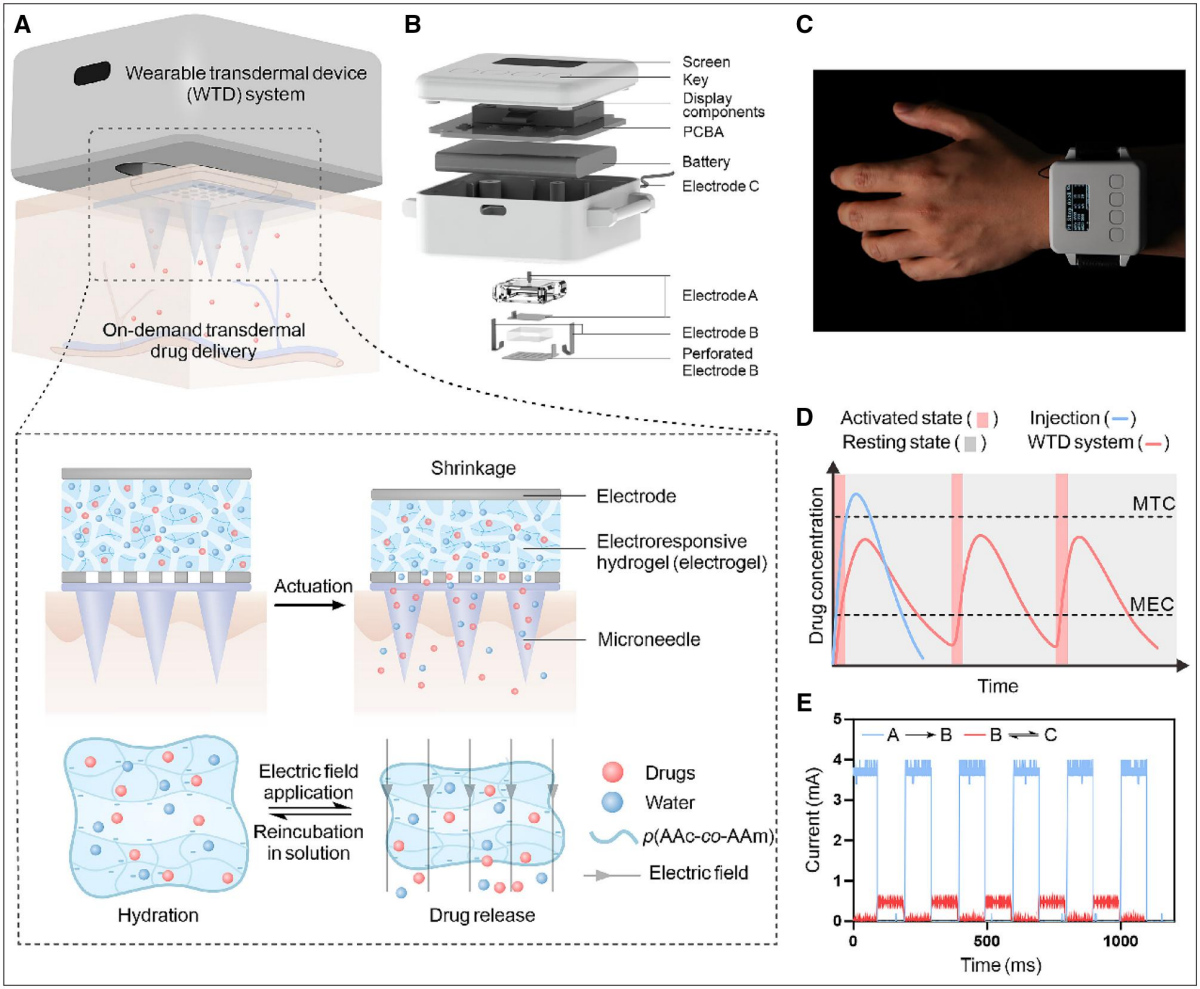
Intelligent materials related to electrical response. (**A**) Diagram of transdermal medication administration using the WTD system. (**B**) Exploded illustration of the respective parts. (**C**) The WTD is positioned on the arm. (**D**) Diagram showing drug concentration profiles following transdermal delivery via injection or with the WTD system. Maximum tolerated concentration (MTC). Minimum effective concentration (MEC). (**E**) Representative output current measured between electrodes a and B (blue) and between B and C (red). Reproduced with permission from Ref. [86]. Copyright 2025 © Elsevier.

Electromagnetic functional biomaterials have great potential in precision oncology. Wang *et al.* [86] developed a wearable transdermal device (WTD), which consists of a mobile ion delivery unit, an electro-responsive hydrogel, and a polymer microneedle patch. The electro-responsive hydrogel is a PAA–acrylamide copolymer [p(AAc-co-AAm)], which carries drugs such as insulin and achieves on-demand transdermal drug delivery. In this study, the synergistic effect of the electro-responsive hydrogel contracting and releasing drugs under electric field stimulation, the enhanced skin penetration of the microneedles and the depth of ion delivery were utilized to accomplish effective and accurate transdermal administration of large-molecule drugs (Figure 5). Recently, a parallel resonant pulsed magnetic field generator has been developed for biomedical purposes, enabling flexible modulation of oscillating magnetic field parameters such as amplitude, oscillation number and frequency, which holds promise for magnetoporation and synergistic cancer therapy [87]. Intelligent responsive materials mainly focus on the ‘switch-like’ response of the materials to external stimuli.

### Bionic functionalized biomaterials

**Figure 6:**
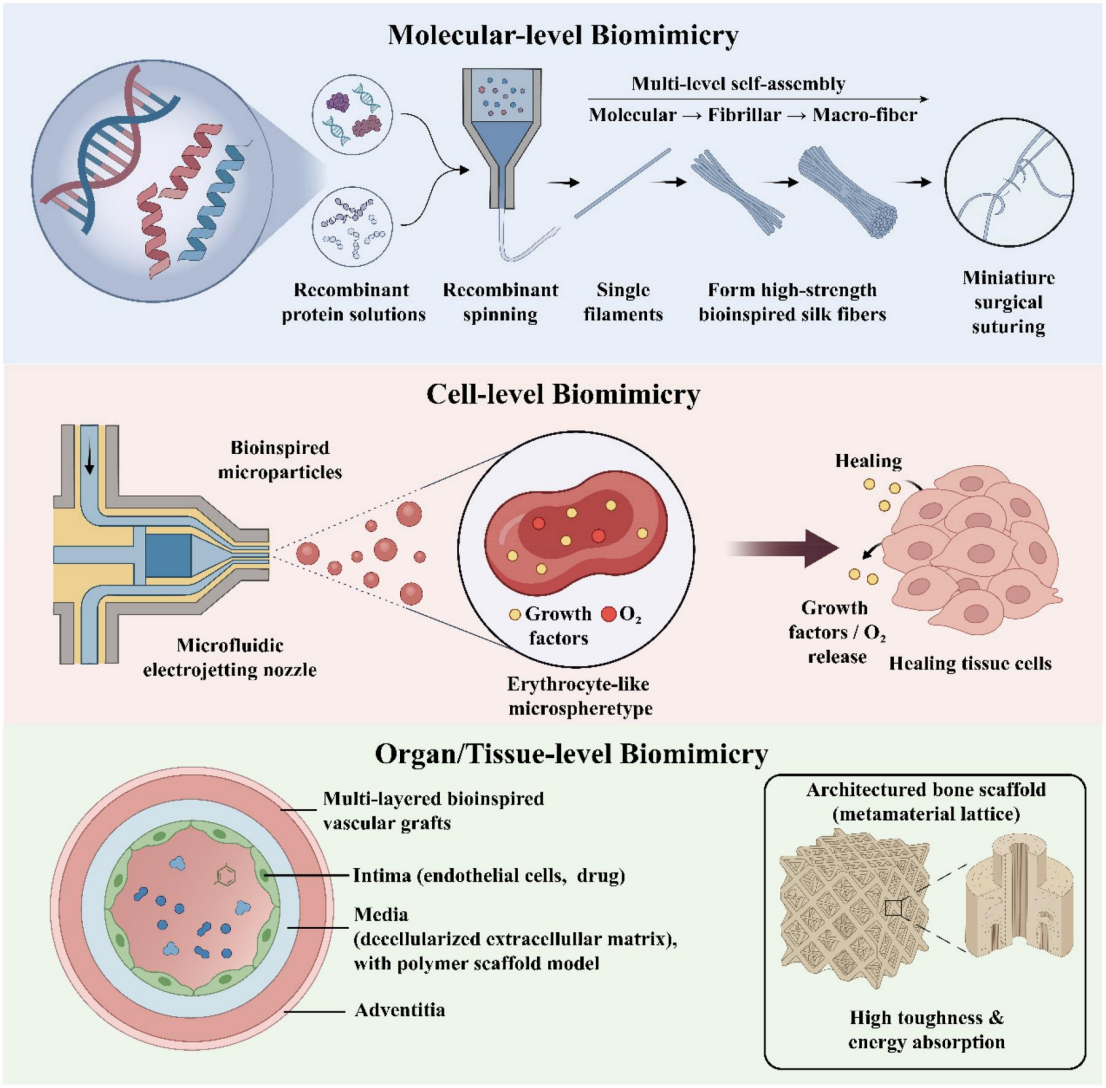
Schematic illustration of bionic functionalized biomaterials across three hierarchical levels: molecular-, cell-, and organ/tissue-level biomimicry.

Bionic functionalized biomaterials are derived from the exquisite architecture and operation of natural biological systems and are of great significance for tissue engineering and regenerative medicine [88, 89]. A core engineering principle of biomimetic functional materials is biomimetic structure and function, aiming to mimic the multiscale architecture, physical properties or biological recognition capabilities of natural tissues [90] (Figure 6).

#### Molecular-level biomimicry

**Figure 7:**
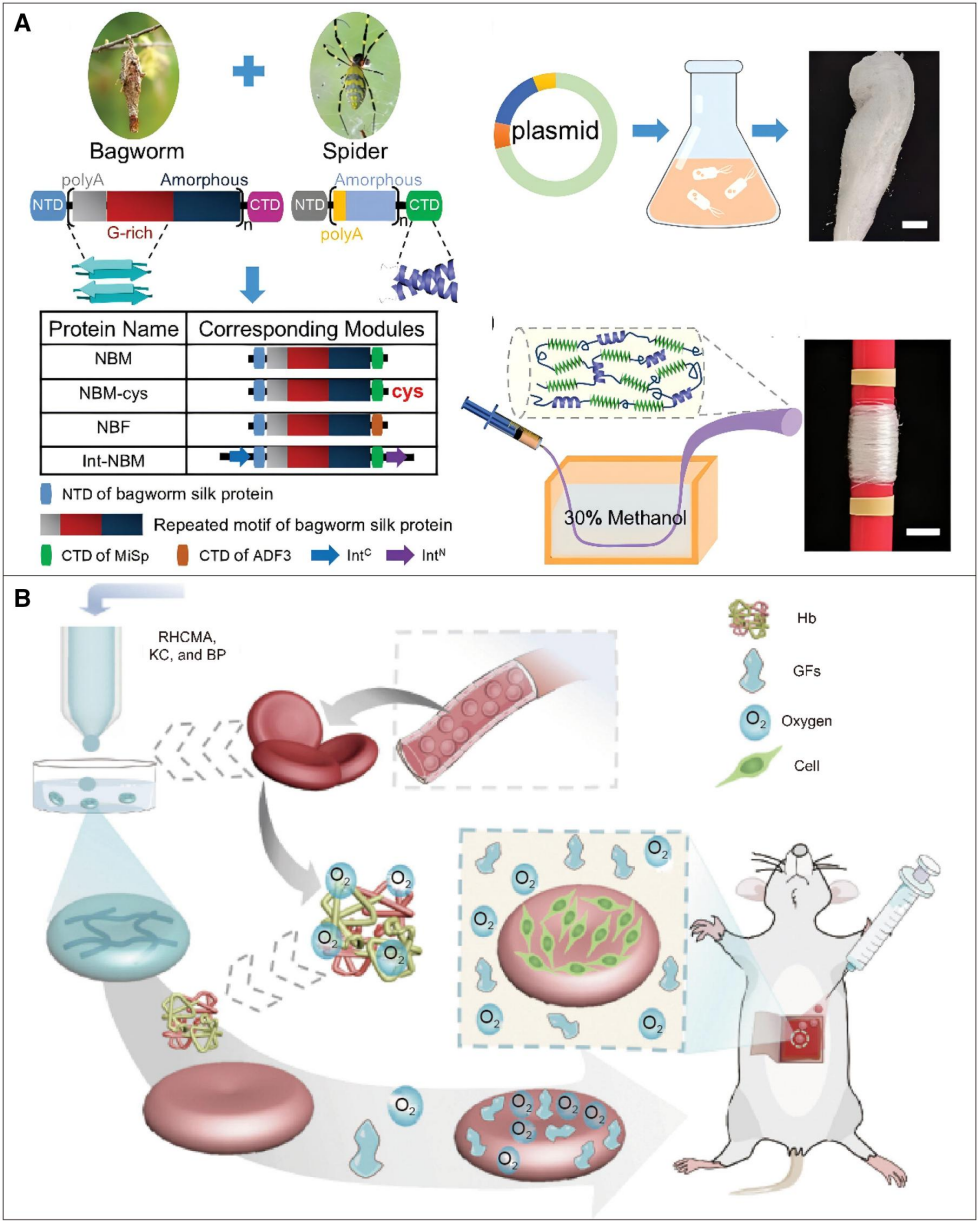
Functionalized biomaterials at the molecular level and cellular-level biomimicry. (**A**) Schematic depicting the rational design and scalable production of chimeric proteins and fibers. Reproduced with permission from Ref. [102]. Copyright © 2024 Wiley-VCH GmbH. (**B**) Schematic depicting the fabrication of ELMPs via microfluidic electrospray, their loading with oxygen and GFs, and their role in promoting damaged tissue regeneration. Reproduced with permission from Ref. [113]. Copyright 2024 © Elsevier.

Molecular-level biomimicry is the cornerstone of biomimetics. It focuses on understanding and imitating the intricate structures, unique functions and dynamic processes of biological molecules and their assemblies to design and create biomaterials and systems with outstanding performance or novel functions [91–93]. Common areas in the field of molecular-level biomimicry include the simulation of biological macromolecules (protein biomimicry, polypeptide biomimicry, nucleic acid biomimicry) [94–97], biomolecular interaction biomimicry [98], biomimetic simulation of the phospholipid bilayer structure of biological membranes [99], biological mineralization biomimicry [100] and so on. In particular, the insect cuticle—with its hierarchical organization, diverse mechanical properties and versatile molecular components such as resilin and cuticular proteins—serves as a rich source of inspiration for biomimetic interface materials [101]. For instance, because natural silk possesses excellent mechanical properties, it has received extensive attention. Zhang *et al.* [102] successfully developed biomimetic silk protein fibers with an elastic modulus of approximately 15 GPa through *in vivo* polymerization and multilevel self-assembly strategies, using genetically engineered silkworm silk proteins and spider silk proteins. Compared to natural spider silk, the fiber demonstrated greater strength. Furthermore, owing to their excellent mechanical performance and outstanding biocompatibility, the fibers hold great promise for biomedical applications, particularly in wound suturing (Figure 7A).

#### Cell-level biomimicry

Cell-level biomimicry is the creation of an engineered system that can perform one or more key cellular functions [103]. It involves extracting the core design principles of cellular functions and implementing them through engineering [104]. For instance, cell membrane biomimetic technology is a method for replicating the characteristics of cell membranes [105]. Through the bioinspired integration of cell membrane characteristics and artificial core functionalities, it significantly enhances biocompatibility and enables long-circulating and targeted delivery within the body [106, 107]. Although nanoparticles encapsulated by cell membranes have obvious advantages, there is still much work to be explored before they can be applied in clinical settings [108].

Biomimetic technology of cell membranes can be applied to drug delivery, tumor treatment, immune regulation, detoxification and other fields [109]. Erythrocytes, the predominant cell type in blood, are characterized by a distinctive biconcave discoid morphology and remarkable deformability [110], which enables them to efficiently transport oxygen and other molecules, promoting cell proliferation and tissue remodeling [111]. During the process of tissue self-repair, inspired by the fact that red blood cells promote cell proliferation and tissue remodeling by providing sufficient oxygen [112], Luo *et al.* [113] developed a novel biomimetic erythrocyte-like microsphere type (ELMP). ELMPs have a bilayered disc shape similar to red blood cells, mechanical flexibility and an exceptionally large specific surface area coupled with stacking stability. These microspheres are fabricated using microfluidic electrojetting technology and can release oxygen and GFs in response to near-infrared light, showing great promise in tissue engineering (Figure 7B).

#### Organ/tissue-level biomimicry

Biomimicry at the tissue/organ level is not merely about replicating a single structure or material but aims to imitate the working principles, structural organization and dynamic behaviors of functional tissues or complete organs composed of multiple cells and extracellular matrices [114]. Bionic structures provide inspiration and innovative solutions for the creation of high-performance, multifunctional and sustainable new materials and structures [115, 116].

**Figure 8:**
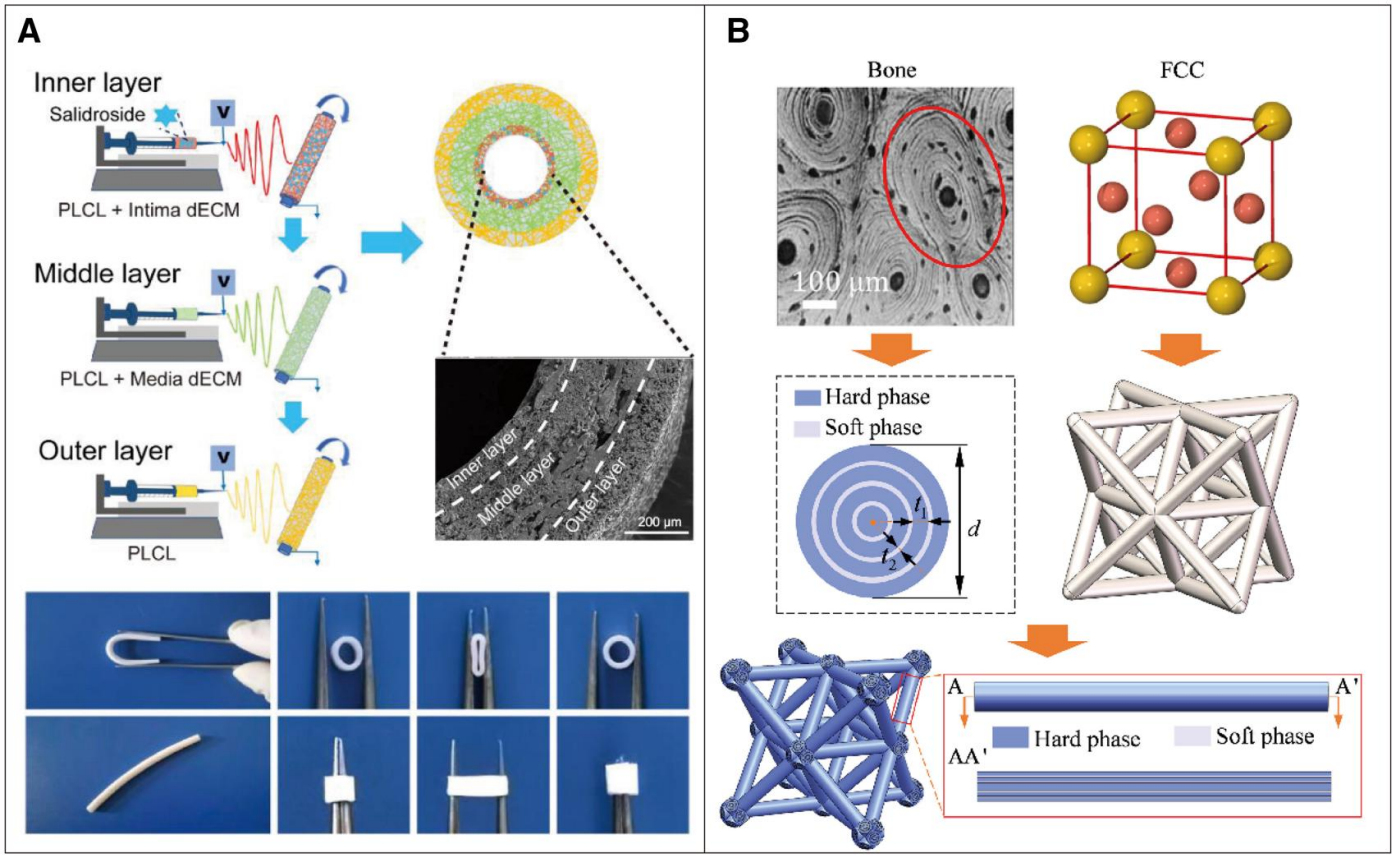
Functionalized biomaterials at the tissue/organ levels. (**A**) Preparation and macroscopic morphological study of the three-layer scaffold. Reproduced with permission from Ref. [118]. Copyright 2023 © Elsevier. (**B**) The design strategy of a multi-bionic metamaterial. Reproduced with permission from Ref. [125]. Copyright 2021 © Elsevier.

At present, the research and application of small-diameter vascular grafts (SDVGs) (<6 mm) face numerous challenges. Tian *et al.* [117] drew inspiration from the natural blood vessels and proposed a simple and efficient fabrication strategy for bionic vascular grafts: using plant-derived regenerated cellulose (RC) hydrogel as the framework, mimicking the extracellular matrix structure of natural blood vessels, and a photopolymerized poly(hydroxyethyl methacrylate) as the filler. This bionic vascular graft avoids the risks of thrombosis caused by the poor elasticity, hydrophobic surface and single structure of the currently used clinical materials. Shi *et al.* [118] developed the three-layer biomimetic SDVG based on poly(L-lactide-co-caprolactone) (PLCL) and porcine arterial decellularized extracellular matrix (dECM) and encapsulated salidroside within the inner layer to prevent thrombosis. They removed the intima, media and adventitia layers of the porcine aorta and used them as the inner, middle and outer layers of the vascular graft. After implanting this scaffold into the rat abdominal aorta, the results showed that the fabricated three-layer vascular graft possesses excellent regenerative ability, which provides a highly promising method for the design and construction of self-expanding vascular stents (Figure 8A). Bionic vascular grafts should not only imitate the static anatomical structure of natural blood vessels but also replicate their dynamic biological functions at all layers. By deeply integrating materials science, engineering and biology, the primary objective is the development of “living”’ vascular grafts that achieve complete host integration, remain open for a long time, and even have growth potential.

Flexible bioelectronic materials are dedicated to developing electronic systems that can seamlessly integrate with biological tissues to monitor physiological signals and provide intervention treatments [119]. Nevertheless, the mechanical disparity between flexible electronic implants and the surrounding biological microenvironment may precipitate an immunological reaction [120]. Among them, biomimetic structure design offers an effective solution by mimicking the mechanical and structural characteristics of biological tissues to enhance biocompatibility. A super-tough and electro-conductive composite, designed to mimic natural tendon for advanced robotics, was reported by Pan *et al.* [121]. A remarkable combination of toughness (420 MJ/m³) and conductivity (1077 S/cm) was achieved in this material by incorporating spider silk, single-walled carbon nanotubes and PEDOT : PSS. Its ability to retain conductive stability over 40,000 deformation cycles meets the critical requirement for long-term functionality, as demonstrated by its use in a humanoid robotic finger for precise grasping.

The optimization of mechanical properties in materials has been contingent upon compositional adjustments [122]. Nevertheless, the inception of mechanical metamaterials has circumvented inherent limitations [123, 124]. Mechanical metamaterials fall under the broader class of man-made architectures that tailor their mechanical properties by changing their geometries and microstructures. Wei *et al.* [125] proposed an innovative multi-bionic strategy combining a face-centered cubic configuration and the concentric circle structure to mimic the crystallographic arrangement of metal atoms and the complex hierarchical structure of bone. This synergistically integrates the high toughness characteristic of bone’s structural organization, significantly enhancing the toughness of the metamaterial and its ability to absorb energy.

Biomimicry at the tissue/organ level serves as a bridge connecting biology and cutting-edge engineering (Figure 8B). This field is moving from imitating morphology to imitating functions and eventually towards creating biomimetic systems with life-like characteristics or surpassing natural functions.

## Technology platform for the preparation and characterization of intelligent and biomimetic materials

**Figure 9:**
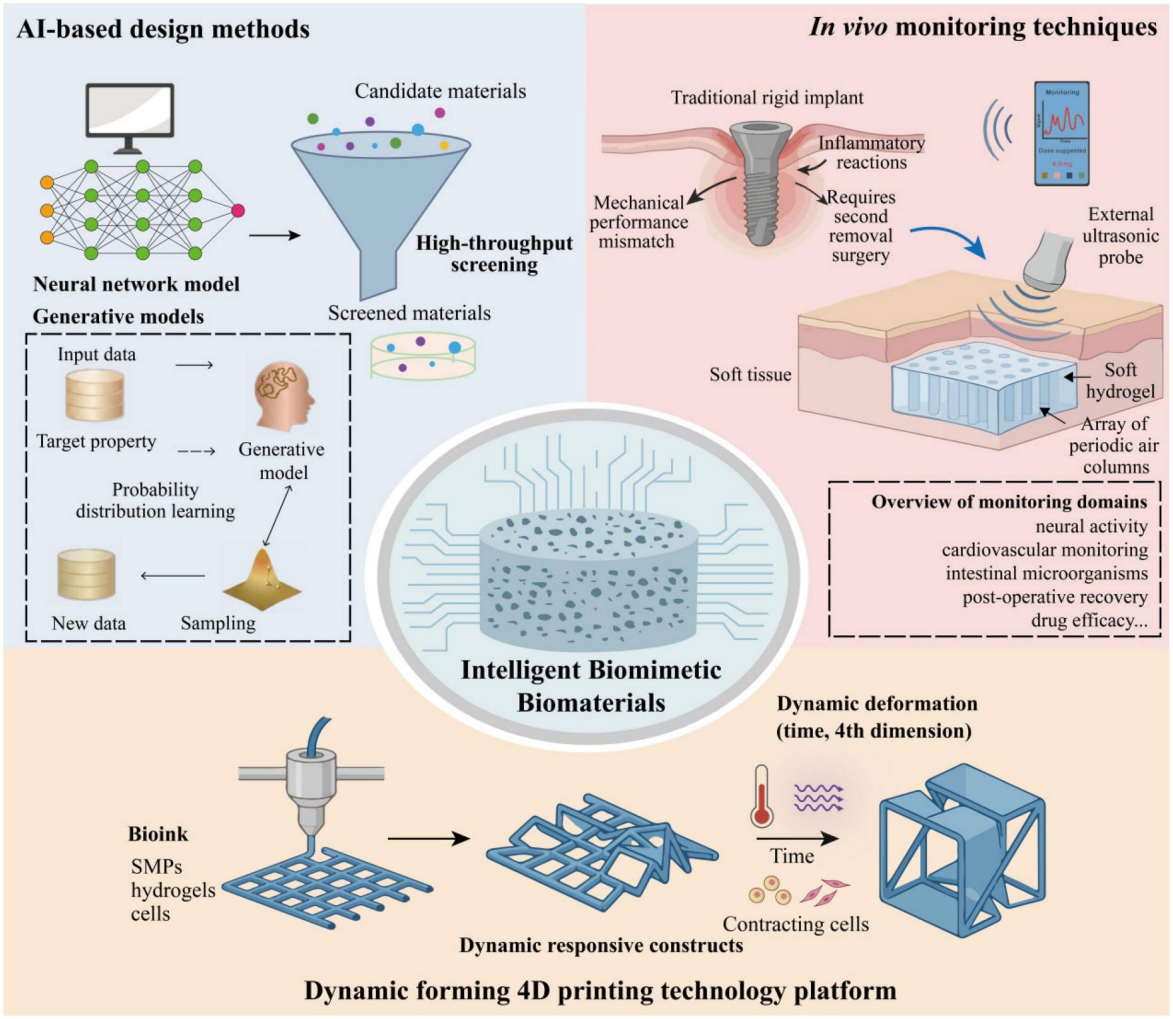
Schematic diagram of the intelligent biomimetic material preparation and characterization technology platform, including dynamic forming 4D printing technology platform, AI-based design methods and *in vivo* monitoring techniques.

The preparation and characterization of intelligent and biomimetic materials are achieved through multiple technological platforms. Here, we introduce three such platforms, including 4D printing for dynamic molding, AI-driven rapid design screening and *in vivo* real-time monitoring. Together, they form a closed-loop system from intelligent manufacturing to adaptive *in vivo* regulation **(**Figure 9**)**.

### 4D printing and precise forming technology

The 4D printing was first introduced in 2013 and immediately attracted great attention from various research fields [126]. 4D printing is conceptualized as a process that employs “programmable materials” via 3D printing to fabricate structures capable of autonomously modifying their physical properties when triggered by predefined signals [127]. Programmable materials essentially refer to intelligent materials that can undergo programmable shape recovery, deformation or actuation in response to external stimuli [128]. The main material categories currently include SMPs, liquid crystal elastomers (LCE), stimulus-responsive hydrogels and shape memory alloys (SMA). The 4D-printed biological materials can respond to internal or external stimuli and perform deformation in a programmed manner, thereby achieving precise matching in both time and space with the dynamic biological microenvironment that changes during the tissue regeneration process [129]. In terms of material mechanisms, dynamic deformation can be achieved through various physical and chemical pathways such as expansion difference [130], shape memory effect [131], degradation kinetics [132] or cell mechanics [133]. Each of these mechanisms determines the speed, amplitude and reversibility of the deformation. Cao *et al.* [130] reported a custom-designed 4D-printed biocompatible hydrogel expander. Using digital light-curing technology, they synthesized water-soluble polymer sheets with a nonswelling elastic framework. After absorbing tissue fluid, these sheets would bend, achieving anisotropic and programmable deformation, with time as the fourth dimension, forming the pre-designed 3D structure. The initial thickness of this expander was only 1.0 mm, significantly thinner than previous devices (3–5 mm), and could be minimally implanted into the rat scalp. Five days after implantation, the skin area expanded to twice its original size, the weight increased to three times its original amount, and there was no tissue damage, indicating that this bending-based strategy has a safer and faster expansion effect compared to traditional devices. This research provides a new strategy for personalized regenerative medicine.

A core engineering principle of biomimetic functional materials is biomimetic structure and function, aiming to mimic the multiscale architecture, physical properties or biological recognition capabilities of natural tissues [134]. Additive manufacturing (AM) is the fundamental technology of 4D printing. 4D printing technology has effects within tissue engineering and regenerative medicine that no other technology can match.

A wide range of intelligent materials is used in the field of 4D printing [135]. A key characteristic of such materials resides in their ability to respond to outside triggers and undergo pre-programmed deformations or performance changes [127]. For example: SMP, LCE, hydrogels, SMA and so on [136–138]. Here, SMP is typically composed of a polymer network and has two distinct states: one is a readily manipulable temporary shape, and the other is a permanent shape that the material will regain after activation [139]. The unique behavior observed in this case arises from the reversible transition that occurs between the glassy and rubbery phases of the material, facilitating extensive deformation and shape recovery. Through 4D printing, You *et al.* [140] successfully synthesized a multiresponsive double-layer deformable membrane, the architecture of which comprises SMP and hydrogel layers. By virtue of its responsive surface microstructure, the SMP layer can precisely orchestrate the switch between proliferation and differentiation stages, thereby enhancing bone formation. The incorporation of a hydrogel layer confers upon the membrane the capacity to dynamically and digitally tune its 3D architecture, ensuring precise conformity to the patient-specific bone morphologies encountered in clinical practice. 4D printing has extensive applications in the realm of biomedicine: developing intelligent medical devices, constructing targeted drug delivery platforms, constructing tissue engineering scaffolds, and others [141].

4D printing imposes more stringent multiple requirements on intelligent or stimulus-responsive materials [142]. There is a complex trade-off relationship among printability, biocompatibility, mechanical properties and stimulus responsiveness [143]. The bio-ink must meet the basic requirements of printability, including appropriate shear-thinning behavior, viscoelastic regulation and rapid gelation ability [144]. Protein-based bio-inks such as gelatin methacryloyl (GelMA) have advantages in regulating rheology and crosslinking kinetics, but their mechanical strength is often insufficient [145, 146]. Synthetic polymers such as polyurethane perform well in mechanical properties, but their biocompatibility needs to be regulated [147]. Biocompatibility and degradation rate are the primary factors determining the selection of materials, and mechanical stability needs to simultaneously maintain the shape during the printing process and the tolerance to the physiological environment after implantation [148]. Currently, the evolution of 4D printing materials reveals multiple emerging trends. The current research is no longer satisfied with a single stimulus response. By incorporating multiple nanofillers into the SMP, various driving methods such as heat, light, electricity and magnetism can be achieved, significantly enhancing the environmental adaptability and functional flexibility of the devices. Through multimaterial printing technology, by combining driving materials (such as SMP and LCE) with sensing materials and structural support materials, ‘intelligent structures’ that integrate driving and sensing functions are created [135].

4D bioprinting mainly relies on exogenous physical or chemical stimuli to achieve deformation, thereby ignoring cell mechanobiology [133, 149]. The morphogenesis and remodeling of natural tissues are essentially cell-mediated processes—cell contractile forces (CCFs) drive tissue folding, indentation and elongation and are the core mechanisms for tissue functionalization and specialization [150, 151]. The research by Wang *et al.* [133] made a breakthrough exploration in this regard, developing a composite bioink composed of oxidized methylcellulose alginate (OMA), GelMA and gel microspheres, enabling independent 4D bioprinting driven by CCFs. This study aims to enable the material system to shift from a rigid mechanical condition to a pliable one, permitting CCFs to bring about substantial gel contraction. This research reveals the potential for a shift from exogenous stimulus dependence to endogenous cell-driven forces, but it also exposes the systematic deficiencies in current 4D bioprinting in integrating the cell’s mechanical initiative. CCFs are limited in magnitude and can only function within specific material systems, and the precise pre-programming of deformation direction and amplitude remains difficult.

Although 4D bioprinting has demonstrated remarkable potential *in vitro* and in animal models, its feasibility for clinical translation still faces multiple obstacles [152]. The biocompatibility of intelligent materials requires rigorous *in vitro* and *in vivo* validation [153]. However, many material systems with excellent response performance still have uncertainties in terms of biological safety, especially in terms of the toxicity of degradation products after long-term implantation, immunogenicity and mechanical stability during deformation [154–156]. Additionally, key issues such as the matching of degradation rates of materials with the rate of tissue regeneration and the persistence of functional maintenance after deformation have not been resolved [129, 157]. Moreover, the introduction of dynamic changes in the time dimension by 4D-printed implants poses unprecedented challenges for regulatory agencies [158]. The approval framework for traditional medical devices is based on the quality control paradigm of static products, while 4D-printed products will undergo preset morphological or functional changes after implantation [159]. The long-term performance and safety assessment of 4D-printed products requires the establishment of new testing protocols and standards [158].

**Figure 10:**
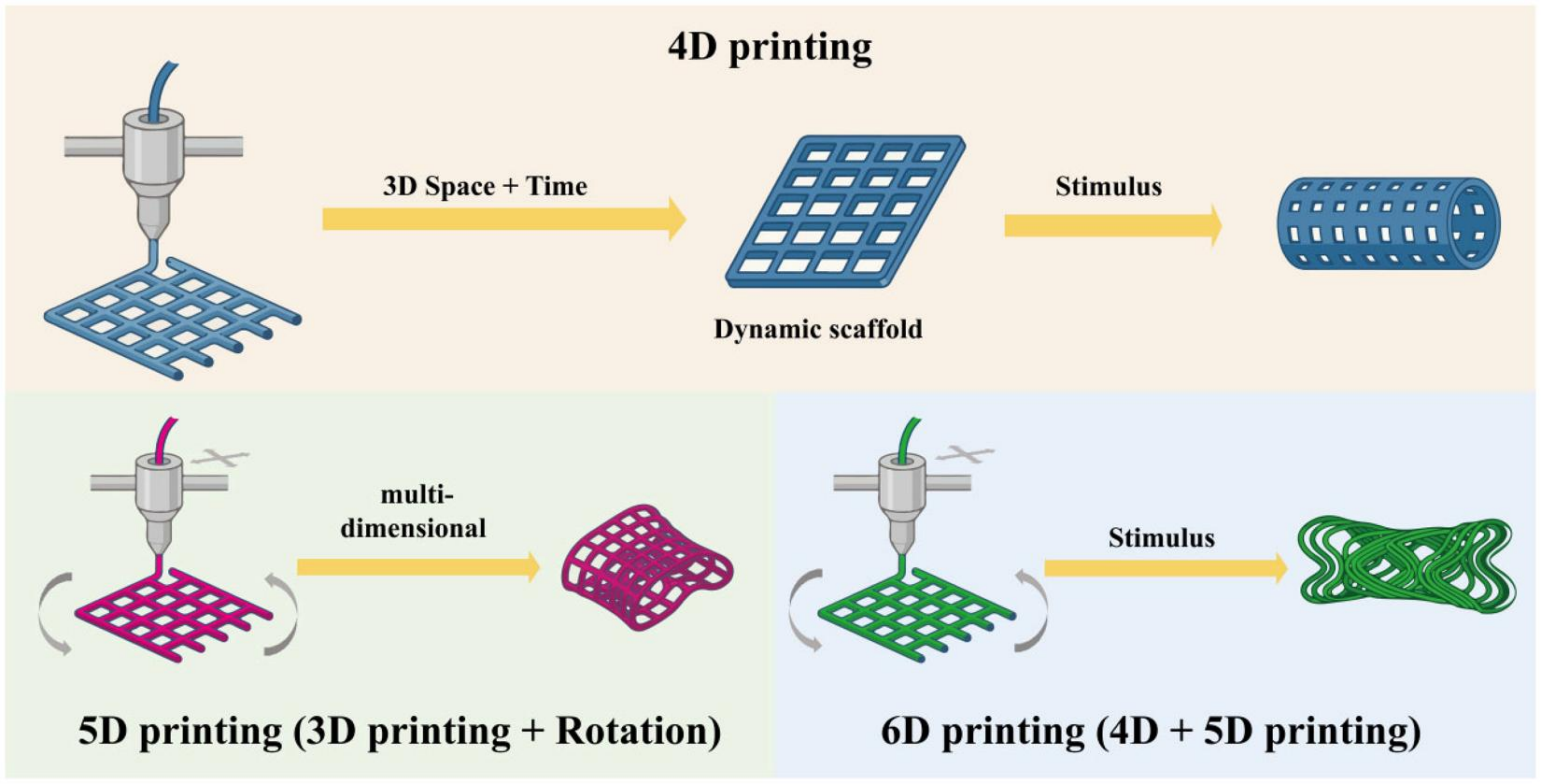
Schematic diagram of the printing process for 4D printing, 5D printing and 6D printing.

Bioprinting technology is currently experiencing a revolutionary advancement. 5D and 6D bioprinting, representing a theoretical extension of 3D printing, have become innovative technologies. 5D printing is executed on a five-axis kinematic system; in addition to the traditional *X*, *Y* and *Z* axes, there are also two more axes [160]. The printing head possesses translational capability, whereas the printing bed can be oriented at a predetermined angle via rotation, which enables the printed object to have multidimensional structures, especially curved layers. Building on the concepts of 4D and 5D printing, 6D printing also focuses on creating stimuli-responsive structures, thereby enabling more precise manufacturing with structural integrity and intelligence [161, 162] **(**Figure 10**)**.

### AI-powered material design platform

The rapid integration of AI and biomedical engineering marks a transformative era for healthcare and materials science [163]. The traditional material development process is lengthy and costly, and it relies on experience and chance. AI data-driven methods have become a key tool for accelerating research and development in biomedical polymers and drug delivery systems [164–167]. These computational techniques enhance the capabilities of traditional methods, enabling scientists to explore a wide range of design spaces, optimize materials and predict previously challenging complex behaviors [167].

**Figure 11:**
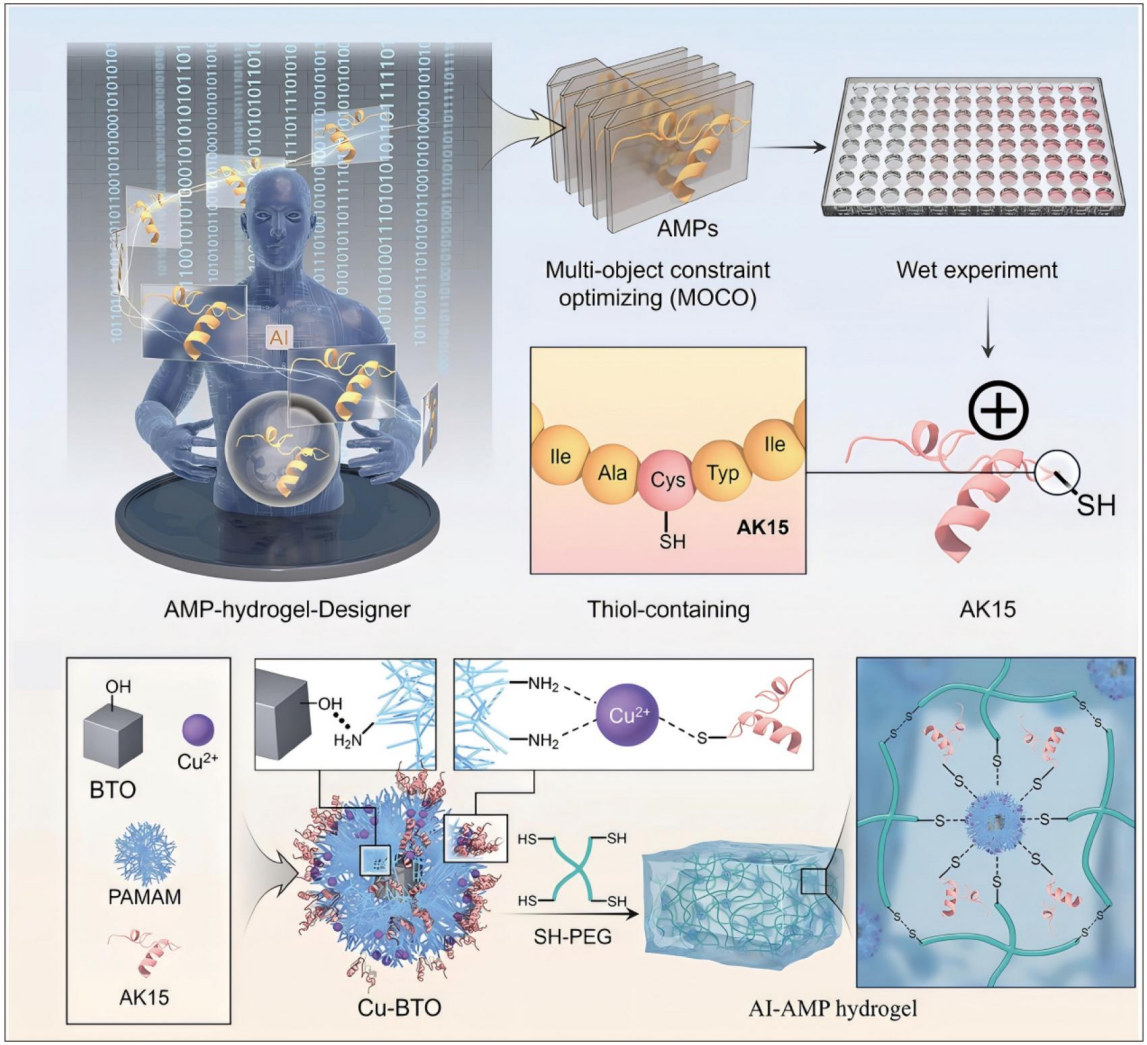
AI-powered material design platform. AI-guided design and performance diagram of AI-AMP hydrogel. Reproduced with permission from Ref. [172]. Copyright 2025 © Wiley-VCH GmbH.

At present, AI has demonstrated its potential in multiple fields, including applications in biomedical polymers and drug delivery platforms, along with material design, characterization and optimization [168, 169]. Moreover, AI is also used to optimize 3D/4D printing, wearable sensors and intelligent materials development [170]. For instance, the myosin proteins and spider silk proteins found in nature, through their intricate β-pleated sheet structures and efficient hydrogen bonding interactions, exhibit outstanding mechanical properties and structural stability, providing important inspiration for the design of artificial proteins. Zheng *et al.* [171] established a computational design platform that combines AI prediction with molecular dynamics verification. Using this platform, the team successfully designed a new protein, ‘SuperMyo’, whose hydrogen bond network was significantly optimized. It surpassed the natural template in both mechanical strength and thermal stability, establishing a robust basis for the creation of novel biomaterials designed for extreme environments. Jiang *et al.* [172] developed an intelligent design platform (AMP-hydrogel-Designer), which is the world’s first AI model based on a deep understanding of the interaction between biomaterials and bacteria. From model construction to peptide synthesis verification, this platform completed the work that traditional methods would take months or even years to accomplish in just 16 days. It can precisely design biomaterials with strong antibacterial activity and achieve a 99.9% antibacterial effect (Figure 11).

In terms of material screening, the AI-enabled high-throughput strategy has significantly accelerated the process of discovering target materials [173]. Yue *et al.* [174] developed a versatile porcine small intestinal submucosa extracellular matrix wound covering coupled with AI for screening antibacterial peptides. Through the AI-optimized hexapeptide CRRI6, it achieved sustained release kinetics for over 6 h, with clearance rates of over 90% against *Escherichia coli* and *Staphylococcus aureus*. This study demonstrated the practical efficacy of AI-assisted antibacterial peptide screening in the design of multifunctional biomimetic dressings.

Generative models are one of the core technologies driving AI-based material design at present [175]. Unlike the traditional high-throughput screening strategy, generative models can directly generate new material structures with the desired properties by learning the underlying distribution patterns of existing material data, achieving a fundamental paradigm shift from forward prediction to reverse design [175, 176].

Representative generative models include generative adversarial networks (GANs), variational autoencoders (VAEs) and diffusion models [175]. Different generative model architectures have their own focuses in material design. The VAE encodes the input data into a distribution within the latent space through the encoder and then reconstructs the data from this distribution through the decoder. Its advantage lies in the good continuity of the latent space, which is convenient for structural interpolation and directional optimization, and is suitable for scenarios that require smooth exploration of the design space [177]. The GAN is trained through the game between the generator and the discriminator and can generate highly realistic samples. It is suitable for generating material systems with complex microstructures [175]. The diffusion model generates the target structure by gradually denoising from pure noise [178]. In recent years, it has demonstrated excellent performance in molecular and crystal generation; for example, the stable probability of generating materials by MatterGen is twice that of traditional models [179].

The integration of generative models with advanced manufacturing technologies represents an important direction in the design of intelligent biomimetic materials [180]. Lee *et al.* [180] developed a moss-like biomimetic thermal-responsive microneedle platform, which integrates three design concepts of motion biomimetics, surface biomimetics and functional biomimetics through AI-guided 4D printing technology. The team used various machine learning algorithms to quantitatively model the complex thermal response behavior of SMPs, with the prediction accuracy of the Gaussian process regression model reaching *R*^2^ > 0.99, providing a reliable confidence interval for the optimization of manufacturing parameters. The resulting microneedle system can simulate the curling and grasping movement of moss leaves, achieve self-driven wound closure and realize antibacterial functions through the continuous DNA release from zinc nanolayers, significantly promoting epithelial regeneration and neovascularization in diabetic wound models. This research fully demonstrates how generative model-driven biomimetic design can span the entire chain from molecular design through mesostructure to macrofunction, achieving truly intelligent biomimetic materials.

The macroscopic properties of materials fundamentally depend on their atomic-scale microstructure and the interaction relationships among atoms. Graph neural networks (GNNs), as a natural fit model for graph-structured data, with their unique message-passing mechanism and relationship learning capabilities, are becoming a crucial computational bridge connecting the microstructure of biomimetic materials with their macroscopic properties. In the design of intelligent biomimetic materials, GNNs have been widely applied to the structural-performance mapping of polymers, porous materials, metamaterials and protein systems, significantly accelerating traditional multiscale computations. In the field of nanobody design, researchers have developed a computational method that combines GNNs with Metropolis Monte Carlo sampling, which is used for the ab initio design of nanobodies [181]. By constructing a GNN model to predict the binding affinity between the nanobody and target protein, and in collaboration with the Monte Carlo sampling algorithm, a systematic optimization of the nanobody sequence and structure was achieved. This method established a physics information-driven deep learning approach, providing a new strategy for the development of protein therapeutic agents.

In terms of performance prediction, AI models have achieved highly accurate predictions of the key performance of tissue engineering scaffolds. Bhaw-Luximon *et al.* [182] studied 15 series of biomimetic nanofiber scaffold families, covering various polymer combinations such as polyesters, polysaccharides and polyester ethers. They used machine learning to predict the macrophage inflammatory response induced by nanofiber scaffolds, and the random forest model achieved the best prediction performance of 92.8% with tumor necrosis factor-alpha (TNF-α) as the output. The deep learning model further demonstrated the potential of convolutional neural networks in classifying macrophage phenotypes based on scanning electron microscopy (SEM) images. At the same time, this study used graph theory analysis to reveal the high similarity in connectivity patterns between electrospun membranes and extracellular matrix, indicating that the scaffold engineering strategy assisted by machine learning and quantified by graph theory is expected to systematically predict cell interactions. Integrating AI into the design of biomaterials has facilitated the creation of responsive and predictive systems capable of dynamically responding to the physiological needs of the skin, including various skin treatment scenarios like wound healing, tissue engineering and drug delivery [183]. Meanwhile, deep learning and federated learning can improve the effectiveness of wound care systems through data-driven healing trajectory prediction and personalized intervention [184].

AI is driving a fundamental transformation in synthetic biology sensors (SBBs) from traditional rational design to AI-driven predictive engineering. Synthetic biology constructs two major synthetic biology sensing platforms, cellular and cell-free, through modular biological components and programmable genetic circuits, achieving functions such as multitarget detection and signal amplification. However, it still faces core challenges such as high design complexity, high engineering costs, cell noise interference, large batch-to-batch variations and difficulties in processing high-dimensional data. The massive data processing, pattern recognition and complex system optimization capabilities of AI can fundamentally solve the development bottlenecks of traditional SBBs, enabling full-chain empowerment from sequence-function relationship prediction to performance iterative optimization. Zheng *et al.* [171] constructed a seamless computational design loop by maximizing hydrogen bonding. First, in the structure generation step, a new backbone with an extended β-sheet chain was generated using the natural myosin I27 as a blueprint through the diffusion model. Second, in the sequence design step, a reverse-folding approach was used to design the amino acid sequence that could precisely fold into the target backbone. Finally, physical screening was conducted, and a rigorous ‘stress test’ was conducted through molecular dynamics simulation to select candidates where hydrogen bonds could coherently break under the stress conditions. This process perfectly integrates the creativity of AI with the rigor of physics, achieving precise directed evolution from chemical principles to protein structure. Through the aforementioned platform, the researchers successfully developed a series of anisotropic super-stable proteins under the code name SuperMyo. In terms of mechanical strength, the denaturation force of SuperMyo-F553 is as high as 1050 pN. When prepared as a protein hydrogel, it remains transparent, elastic and in a complete gel state even after sterilization at 121°C under high pressure. Future biomedical materials (such as tissue engineering scaffolds, *in vivo* implant sensors) will be able to directly adopt the industrial standard of the wet-heat sterilization process, significantly reducing the application threshold and infection risk.

### In-body monitoring technology

To achieve safe, long-term and precise in-body monitoring, the primary issue to be addressed is the biocompatibility between the materials and the dynamic physiological environment. Traditional rigid implants can cause inflammatory reactions due to mechanical performance mismatch [185]. The new generation of biomaterials should be compatible with the physiological environment. For applications such as post-operative monitoring, the in-body monitoring materials should also be biodegradable, owing to their hydrolysis into nontoxic, absorbable substances; these materials spare patients from undergoing a second removal surgery [186, 187]. Currently, the in-body monitoring material technology has been extensively studied in the monitoring of neural activity, cardiovascular monitoring, monitoring of intestinal microorganisms and chemical environments and post-operative recovery and drug efficacy monitoring [188–191].

**Figure 12:**
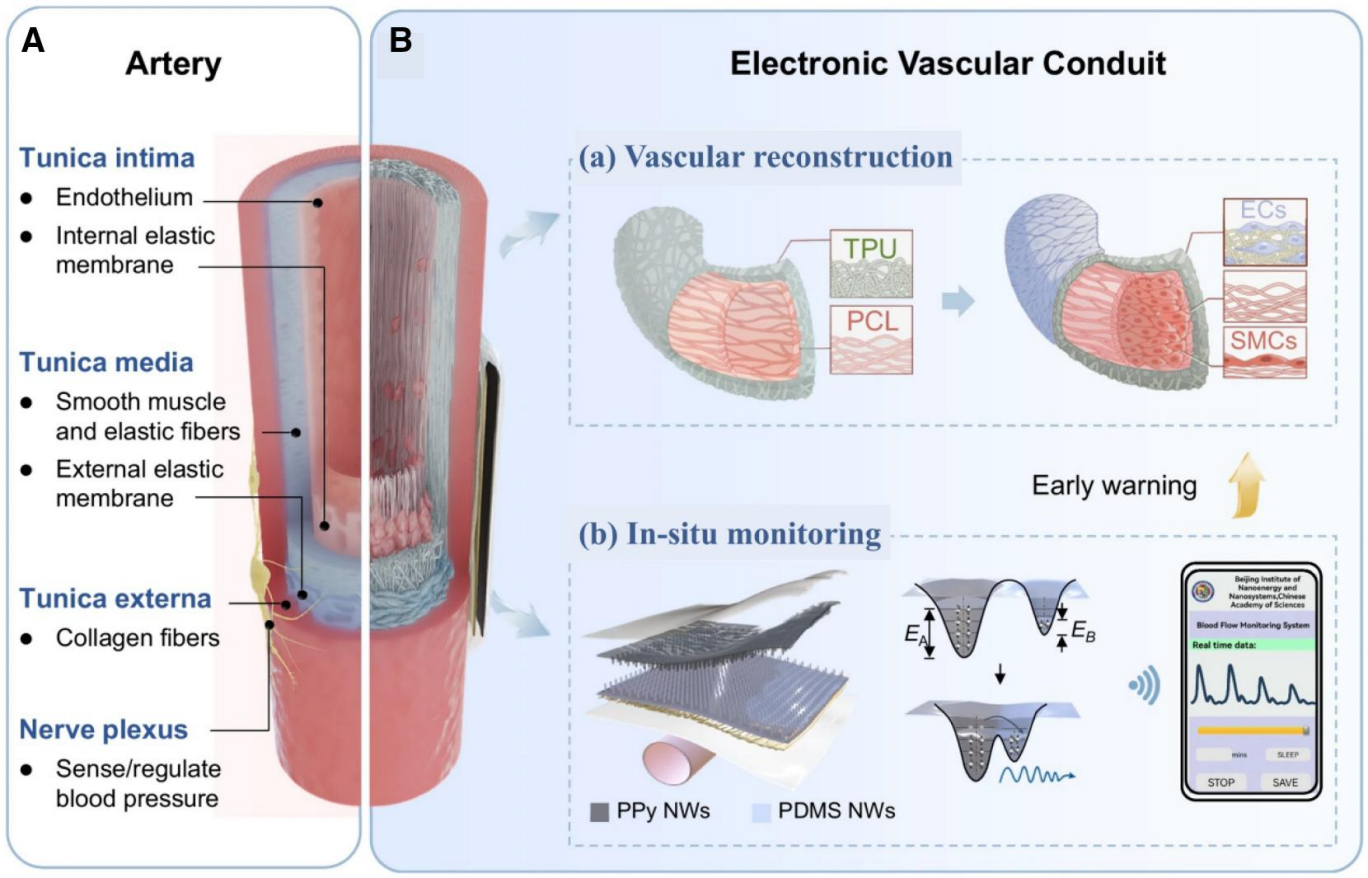
Overview of the native artery and electronic vascular conduit. (**A**) The composition of the native artery. (**B**) Our electronic vascular conduit comprises (a) a biomimetic artificial graft and (b) an integrated triboelectric sensor. Reproduced with permission from Ref. [195]. Copyright 2025 © Springer Nature.

In the field of cardiovascular monitoring, especially for patients suffering from hypertension, hyperlipidemia and diabetes, the postoperative period carries a lifelong threat of vascular occlusion [192–194]. Based on this, Liu *et al.* [195] pioneered an electronic vascular conduit that seamlessly integrates flexible electronic components into biomimetic vascular grafts, thereby enabling continuous, real-time hemodynamic surveillance in pathological states such as vascular stenosis and thrombosis, while simultaneously facilitating postoperative vascular regeneration. Due to the increased damage to internal tissues and cardiovascular diseases, existing clinical monitoring approaches suffer from shortcomings in achieving accurate strain measurement and sustained monitoring. Although implantable sensors have potential, they face problems such as mismatch with tissue mechanics, poor biocompatibility and degradability and difficulties in wireless monitoring **(**Figure 12**)**. The ultrasonic metagel sensor introduced by Tian *et al.* [196] is a two-dimensional phononic crystal featuring periodic air column arrayed in soft hydrogel, enabling continuous wireless monitoring of tissue strain *in vivo*. Phononic crystal theory forms the basis for the ultrasonic metagel sensor’s functionality. The periodic air column structure inside forms a phononic crystal with a specific acoustic bandgap. When the sensor deforms along with the tissue, the structural parameters of the air columns change, causing a frequency shift in the acoustic bandgap. When the external ultrasonic probe emits ultrasonic waves and encounters the sensor, the peak frequency of the reflected wave changes as the bandgap moves. By analyzing the spectral characteristics of the reflected wave, the strain of the tissue can be monitored.

In the field of *in vivo* monitoring technology, biosensors, as the core enabling component, have expanded from simple physiological parameter monitoring to a systematic technology platform that integrates multimodal sensing, intelligent feedback and adaptive regulation [197]. Implantable biosensors are evolving towards minimally invasive, long-lasting and high-fidelity directions. Zhu *et al.* [198] developed the nano-structured biological analysis microneedle (RNB) platform, which repositioned the electrode function from a simple sensing substrate to a core mechanism for resilient, high signal-to-noise ratio *in vivo* measurement. Through a double-layer gold adhesion layer and a controllable alloying process, a corrosion-resistant wide potential window and wear-resistant nanocavity texture interface were constructed. This platform enables minimally invasive, continuous and real-time monitoring of therapeutic drugs and organ functions.

Furthermore, synthetic biology and tissue engineering can be deeply integrated. Sawayama *et al.* [199] developed an ‘*in vivo* sensor display’ implanted in the skin. By genetically engineering human keratinocyte stem cells, they enabled them to activate enhanced green fluorescent protein (EGFP) expression through the nuclear factor-κB (NF-κB) pathway when sensing inflammatory signals such as tumor TNF-α. After transplantation, it successfully vascularized in mice and achieved stable function for over 200 days. The fluorescence intensity was quantitatively correlated with the degree of inflammation. After inflammation subsided, it could again respond to repeated stimulation, representing a fundamental paradigm shift in biological monitoring from wearing devices to implanting life.

Magnetic resonance imaging (MRI), with its combined advantages of no ionizing radiation, deep tissue reach and high definition, has become one of the preferred visualization modalities for *in vivo* monitoring of intelligent biomimetic materials [200]. However, traditional MRI relies on passive exogenous contrast agents and it is difficult to achieve real-time correlation with cellular activity and enzyme metabolism [201]. Mukherjee *et al.* [202] have developed the modular aquaporin proteinase activation reporting platform, which successfully constructed a universal platform for systematically creating genetically encoded MRI sensors through two independent design strategies: protein stabilization and subcellular transport. This platform enables noninvasive visualization of multiple targets in live mammalian cells, integrating the precision of genetic engineering with the deep imaging capabilities of MRI. It provides a new tool for tracking the fate and biological activity of cells surrounding implanted intelligent biomimetic materials. The deep integration of optical coherence tomography (OCT) and AI has paved the way for the dynamic assessment of intelligent biomimetic materials. The automated cell characteristic analysis toolbox developed by Babaei *et al.* [203] combine OCT and a deep learning-powered segmentation algorithm, achieving an average segmentation accuracy of 88.96%. It enables noninvasive, high-throughput quantitative analysis of cell viability and morphological characteristics (such as elongation rate, flatness and surface roughness) within 3D bioprinted hydrogel scaffolds, providing a powerful computational tool for rapid monitoring of 3D cell cultures and the performance of biomaterials.

Although *in vivo* monitoring technologies offer substantial potential, ensuring long-term functional stability of these complex material interfaces within the body, as well as how to achieve their low-cost and high-consistency manufacturing, remains a major challenge that must be overcome before moving towards clinical trials.

## The frontiers and clinical application progress of intelligent and bionic biomaterials

**Figure 13:**
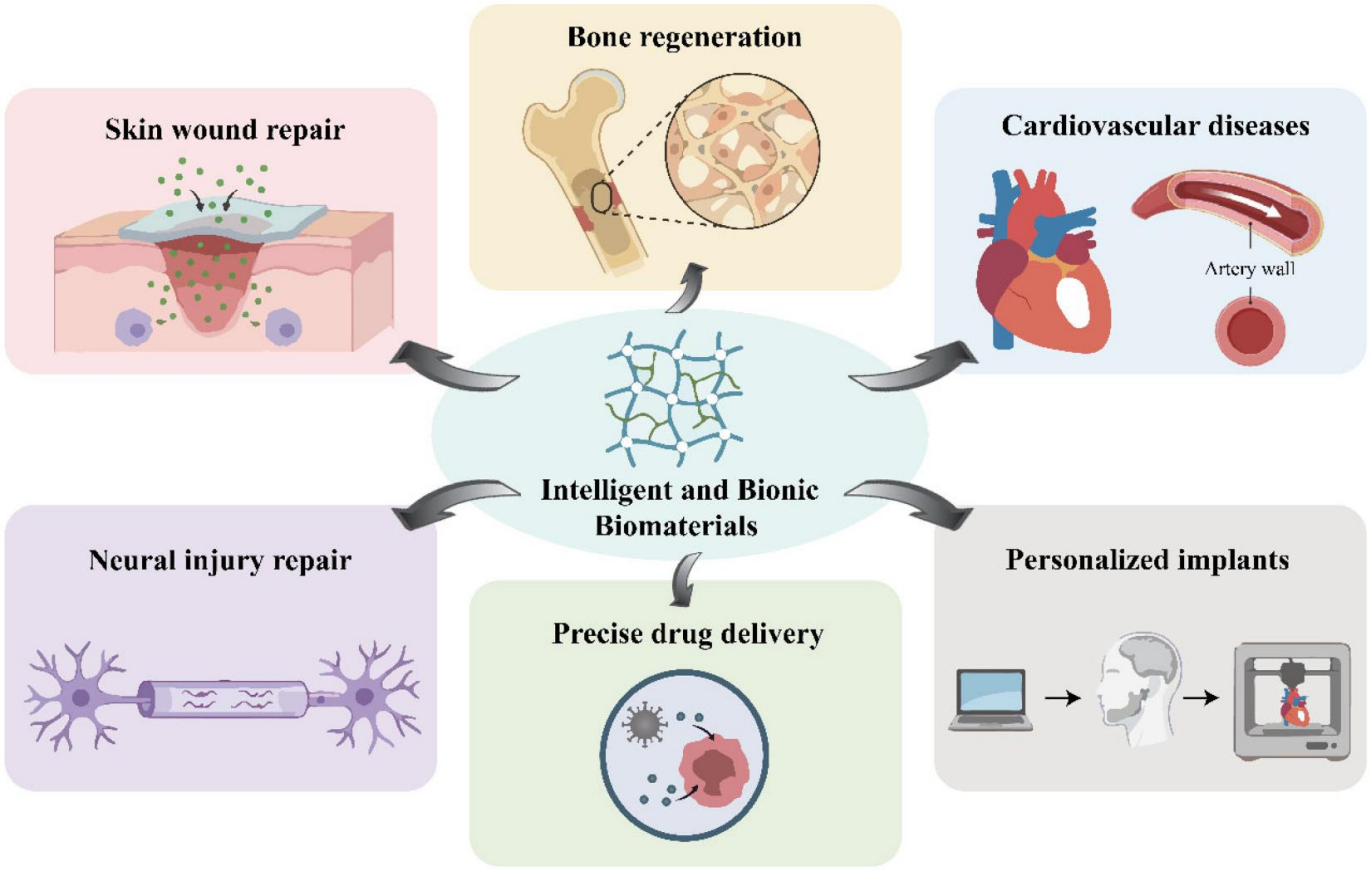
Schematic diagram of intelligent and bionic biomaterial applications. The cutting-edge and clinical applications of intelligent biomimetic materials in areas such as skin wound repair, bone regeneration, cardiovascular diseases, neural injury repair, precise drug delivery and personalized implants.

This section mainly introduces the cutting-edge and clinical uses of intelligent and biomimetic biomaterials in fields such as skin, bones, cardiovascular systems, nerves, drug delivery and personalized implant systems (Figure 13).

### Skin wound repair

The ideal intelligent dressing should be able to sense the biomarkers (such as pH, enzymes, ROS) of the wound during the inflammatory, proliferative [204, 205] and remodeling phases, and make dynamic responses to provide sequential treatments (such as early antibacterial treatment, mid-term vasogenic promotion and late scar prevention) [206]. Wound healing involves a sequence of consecutive stages, including inflammation, cellular migration and tissue remodeling. However, an excessive inflammatory response or dysregulation of regenerative signaling pathways may result in delayed healing and scarring [207]. Yin *et al.* [208] successfully incorporated β3T-curcumin (β3Tc) into UV-crosslinked hyaluronic acid methacryloyl (HAMA) to develop an intelligent hydrogel system (HA-β3Tc) based on tetrahedral framework nucleic acid (tFNA). In this work, they successfully constructed and characterized the multifunctional nanostructure β3Tc and efficiently loaded curcumin. This structure has excellent stability, dispersibility and biocompatibility. At the same time, the system contains a time-controlled mechanism. In the early stage, curcumin is liberated to inhibit the NF-κB signaling cascade and diminish inflammation. In the second phase, TGF-β1 is spatially sequestered to activate fibroblasts and promote collagen synthesis. This temporal regulation and adaptation more closely mimic the natural healing process, allowing rapid, efficient and scarless tissue regeneration. It is a very promising platform technology for the clinical treatment of refractory wounds, including burns, diabetic foot ulcers and chronic wounds.

The process of wound healing is characterized by a highly synchronized sequence of physiological responses. If left untreated in a timely and proper manner, the wound could get infected, induce inflammation and, in extreme cases, advance to tissue damage requiring amputation [209]. Self-powered materials can deliver sustained electrical stimulation via energy harvesting and conversion without depending on external power supplies, thus speeding up wound healing.

**Figure 14:**
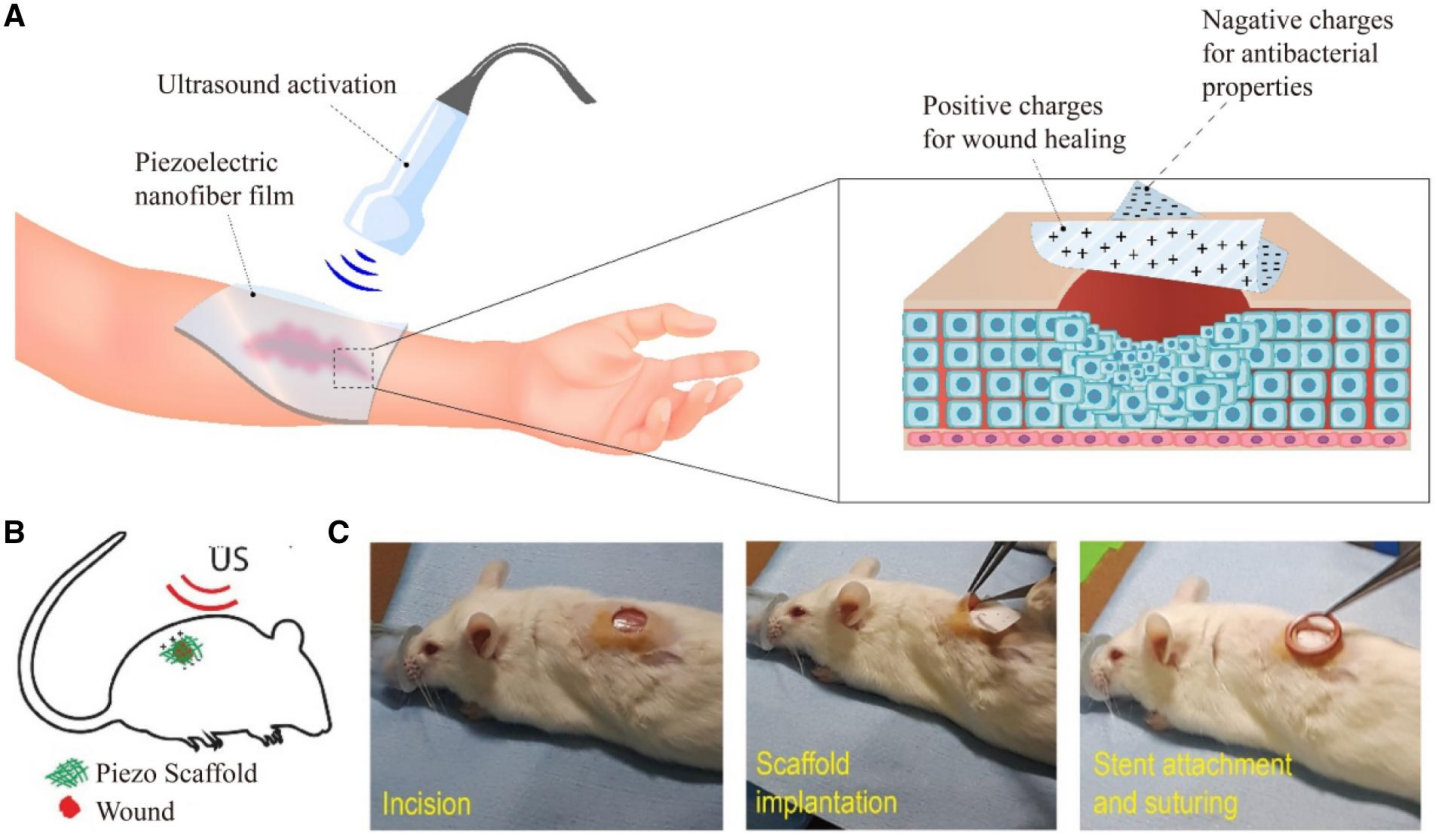
Intelligent materials for skin wound repair. (**A**) Utilization of biodegradable piezoelectric PLLA nanofibers placed on a wound and stimulated externally with US to generate a tunable surface charge, aiming to accelerate wound healing and inhibit bacterial infections. (**B**) Simplified schematic diagram of *in vivo* experiments. (**C**) Sequential presentation of each step of the surgery: a key-sized wound was surgically treated on the mouse, and a nanofiber scaffold was implanted into the site to monitor the tissue repair process. The researchers used US at the wound site of the animal to simulate the *in vitro* system. Reproduced with permission from Ref. [211]. Copyright 2024 © Elsevier.

A plethora of self-powered materials have been developed to assist skin wound healing, such as piezoelectric materials, triboelectric materials, thermoelectric materials, photovoltaic materials, capacitive materials and bioelectric materials (Table 1) [210]. Das *et al.* [211] recently published an innovative degradable piezoelectric skin wound scaffold that employs the poly-L-lactic acid (PLLA) nanofiber scaffold and is triggered by external ultrasound (US) to generate tunable surface charges to perform dual functions of wound healing activity and antibacterial efficacy. Although the existing hydrogel particle platforms can encapsulate and deliver therapeutic agents, most of them are unable to achieve comprehensive treatment that promotes tissue repair after antibacterial treatment (Figure 14). Huang *et al.* [212] proposed a spatial-temporal piezoelectric microcapsule whose outer shell encapsulated potassium sodium niobate, and vascular endothelial growth factor (VEGF) was incorporated into the core. The microcapsule was capable of achieving US-assisted wound infection treatment through microfluidic technology. This provides a new application platform for piezoelectric materials in acoustic-driven sterilization and programmable wound care.

**Table 1:** Category, mechanism, core function and representative materials of self-powered materials.

Category	Mechanism	Core function	Representative materials	Ref.
Piezoelectric materials	Convert mechanical stress or external US into surface electrical charges via force-electric conversion	Generate controllable charges for antibacterial activity and accelerated wound healing	Poly(vinylidene fluoride-trifluoroethylene) [P(VDF-TrFE)], Barium titanate (BaTiO_3_), Potassium sodium niobate (KNN) and PLLA, etc.	[211, 218, 219]
Triboelectric materials	Convert mechanical energy into electrical energy through contact electrification and electrostatic induction	Supply sustained electrical stimulation without external power sources	Polytetrafluoroethylene (PTFE/Teflon), Polydimethylsiloxane (PDMS), Nylon and Aluminum (Al), etc.	[195, 220–222]
Thermoelectric materials	Transform thermal energy into electricity driven by temperature gradients	Realize self-powered electrical stimulation based on body temperature or wound temperature differences	Bismuth telluride (Bi_2_Te_3_), Bismuth selenide (Bi_2_Se_3_), Silver Selenide (Ag_2_Se) and Poly(3,4-ethylenedioxythiophene): poly(styrene sulfonate) (PEDOT: PSS), etc.	[54, 223, 224]
Photovoltaic materials	Convert light energy into stable electrical energy	Provide noninvasive and remotely controlled electrical stimulation for wound repair	Monocrystalline Silicon, Perovskite (e.g., CH_3_NH_3_PbI_3_), P3HT: PCBM (Organic Photovoltaic) and Black Phosphorus (BP) Quantum Dots, etc.	[225–227]
Capacitive materials	Store and release electrostatic energy in a controllable manner	Deliver stable pulsed electrical signals to regulate wound healing	Graphene, Polypyrrole (PPy), Ruthenium Dioxide (RuO_2_) and Polyimide (PI)/Carbon Nanotube (CNT), etc.	[76, 228–230]
Bioelectric materials	Mimic endogenous bioelectrical signals and match the physiological electrical microenvironment	Mediate ordered cell proliferation and differentiation to promote scarless regeneration	Polyaniline (PANI), MXene (Ti_3_C_2_T_x_), Conductive Hydrogel (e.g., PAAm/Gelatin/SA) and Hyaluronic Acid Methacryloyl (HAMA), etc.	[208, 231, 232]

Chronic Treatment of chronic wounds (such as diabetic foot ulcers and venous ulcers) face two major challenges: the lag in monitoring, which makes it impossible to capture hidden pathological conditions such as infection and ischemia in real time; and the difficulty in analyzing exudate, as the secretion of wound exudate is slow, the composition is complex and existing technologies are unable to efficiently collect and accurately detect inflammatory and infection-related biomarkers [213]. Wang *et al.* [213] developed the iCares smart bandage. This research included 20 patients with diabetic foot wounds and venous leg sores. It was the first time that the real-time monitoring capability of the smart bandage was verified in humans. This system can detect key biomarkers such as nitric oxide, hydrogen peroxide and oxygen levels. It can warn 1–3 days before the clinical symptoms appear. The machine learning algorithm’s classification accuracy for the severity and healing potential of the wounds is comparable to that of experienced clinical doctors.

In the field of skin regeneration, one of the earliest and most successful clinical applications of the biomimetic design concept is the Integra® Dermal Regeneration Template. Its double-layer structure precisely mimics the functions of the skin [214, 215]. The upper layer of the silicone membrane acts as a temporary epidermal barrier, while the lower layer of the collagen-glycosaminoglycan porous matrix provides a biomimetic dermal scaffold for cell migration and vascularization [214]. However, it cannot respond to the dynamic wound microenvironment. Therefore, the current research frontier is dedicated to developing the next-generation composite materials that integrate intelligent functions such as microenvironment response, antibacterial properties and angiogenesis based on this biomimetic structure [216]. At the same time, AI is utilized for automatic wound assessment in acute and chronic wounds, personalized treatment decision support and prediction of healing outcomes [217].

### Bone and cartilage regeneration

Bone tissue engineering aims to achieve effective bone repair and reconstruction by developing advanced biomaterials, bioactive factors or cell technologies [233]. In the selection of materials for bone tissue engineering, a thorough understanding of the composition and architecture of native bone tissue is essential, followed by the judicious choice of biomimetic or customizable synthetic biomaterials, including polymers, bioceramics and composites [234]. The material functions as a “signal hub”, capable of sensing both internal and external cues to modulate the delivery of therapeutic agents or its own properties. Piezoelectric materials are a direct expression of the force-electric conversion effect and are a part of a wide variety of devices in modern times [235]. The application of mechanical stress induces a surface electrical charge in piezoelectric materials. Piezoelectric biomaterials are exploited throughout biomedicine for wound healing and regenerative medicine, taking advantage of their natural capacity to convert mechanical strain into bioelectric signals to activate cellular functions [236, 237]. Wu *et al.* [218] fabricated a barium titanate (BT)/silica calcium composite scaffold via photopolymerization with superior piezoelectricity and satisfactory bioactivity. The porous architecture greatly improved the degradation performance and biocompatibility, offering an innovative approach to designing and fabricating bone repair materials. In addition to composite scaffolds, high-temperature air-assisted 3D printing of polyetheretherketone (PEEK) is shown to significantly improve the mechanical characteristics of artificial bone implants by promoting crystallinity and interlayer bonding, with optimal performance achieved at a forming temperature of 240°C [238].

**Figure 15:**
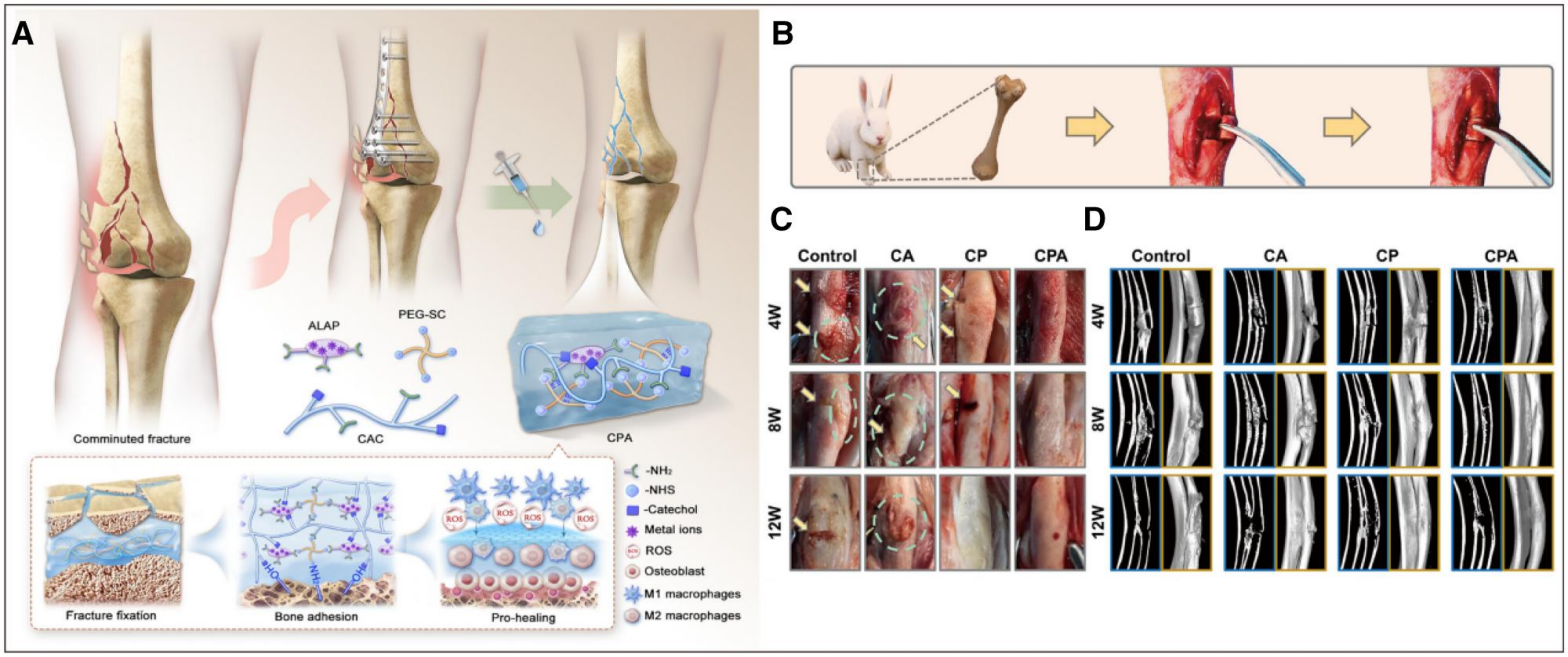
Intelligent materials for bone regeneration. (**A**) Schematic depiction of the fabrication and characterization of the bone adhesive. (**B**) Diagrammatic representation of the rabbit radial fracture model. (**C**) Typical images illustrating fracture healing (yellow arrows point to fracture gaps; green circles highlight ectopic bone formation). (**D**) Coronal sectional views (indicated by the blue frame) and three-dimensional reconstructed images (indicated by the yellow frame) obtained from CT scans at weeks 4, 8, and 12. Reproduced with permission from Ref. [243]. Copyright 2025 © Wiley-VCH GmbH.

Bone regeneration depends on a favorable microenvironment. Therefore, new-generation materials must possess immune regulation and angiogenic promotion functions [239]. By designing the surface chemistry of the materials or releasing specific factors, the initial inflammatory response can be directed towards a pro-repair direction, which is the key to ensuring successful regeneration [240, 241]. Bone defects and nonhealing fractures have always been challenging problems. However, traditional methods such as bone grafting and internal fixation each have their limitations [242]. Cheng *et al.* [243] successfully developed an intelligent bone adhesive (CPA) with immune regulation and bone regeneration functions, offering a novel approach for treating complex fractures and bone defects. Based on the strong adhesion ability of mussels to wet rocks, researchers introduced caffeic acid into the materials. Its unique catechol functional group can generate extremely strong adhesion on wet interfaces, firmly *in situ* fixing the broken bone fragments, providing a stable mechanical environment for subsequent healing and achieving intelligent immune regulation from pro-inflammatory to pro-healing and promoting osteogenesis (Figure 15).

Infuse® Bone Graft is a bone regeneration product based on recombinant bone morphogenetic protein 2 (rhBMP-2). It is one of the most successful commercial cases of growth factor therapy in the field of orthopedics and is an early clinical realization of the intelligent delivery concept [244, 245]. Its core design is to achieve local controlled release of potent osteogenic signaling proteins through an absorbable collagen sponge carrier, thereby achieving active regulation of biological processes [246]. In addition to polymer-based scaffolds, additive manufacturing of biodegradable materials based on zinc has also made recent progress in the field of bone repair. For example, technologies including laser powder bed fusion can produce porous zinc scaffolds with adjustable mechanical properties, degradation rates and good biocompatibility, offering a viable alternative in the treatment of load-bearing bone defects [247].

### Cardiovascular diseases

**Figure 16:**
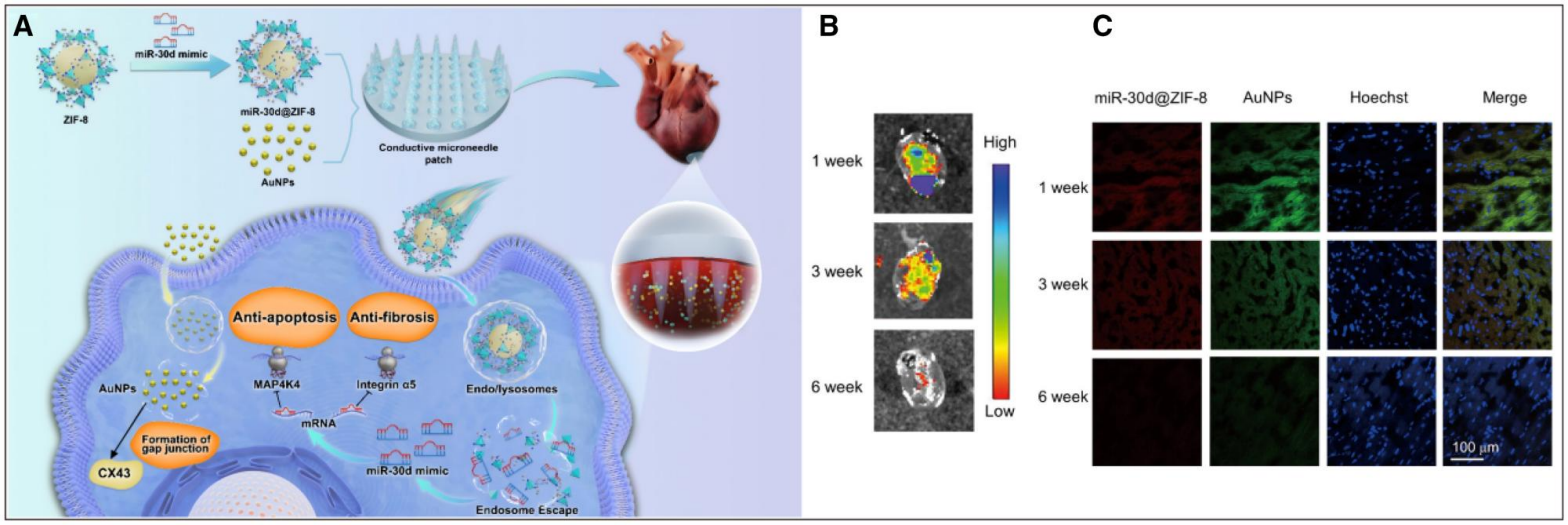
Intelligent biomimetic materials for the repair of cardiovascular diseases. (**A**) The synergistic application of an electroconductive microneedle patch and a miR-30d nanodelivery system confers protection against myocardial ischemia-reperfusion injury (I/RI). (**B**) *Ex vivo* imaging of ischemia-reperfusion (I/R) mice hearts after implantation of a conductive microneedle patch loaded with Cy3-miR-30d@ZIF-8 nanoparticles for 1, 3 and 6 weeks. (**C**) CLSM images of ischemic myocardium from I/R mice after implantation of a conductive microneedle patch loaded with Cy3-miR-30d@ZIF-8 nanoparticles for 1, 3 and 6 weeks. Reproduced with permission from Ref. [253]. Copyright 2024 © American Chemical Society.

Cardiovascular diseases are a primary contributor to global mortality, responsible for millions of deaths each year [248]. The design of cardiovascular materials is shifting from providing mechanical support to dynamic, interactive biological interfaces [249]. In the field of coronary intervention, fully biodegradable stents actively degrade after fulfilling their supporting role, restoring the natural physiological function of the blood vessel [250]. However, the matching of their degradation kinetics with vascular remodeling remains a core issue to be resolved [251]. In the context of tissue repair, the strategic focus of biomimetic vascular grafts lies in guiding the functional reconstruction of host cells in a three-layered “living” blood vessel. Aizarna-Lopetegu *et al.* [252] pioneered an embedded bioprinting paradigm employing hybrid bioinks for the fabrication of multilayer cylindrical artery models. This vascular model is composed of a concentric cylindrical stimuli-responsive layer and a dECM-based layer. The outer layer of the vascular model has been developed with a thermosensitive elastic hybrid ink, which can provide the model with responsiveness and offer structural support to the adjacent inner layer containing cells. By irradiating with near-infrared light, the photothermal response characteristics of the model were observed, demonstrating its potential in biomedical research. Ischemia-reperfusion injury (I/RI) is a crucial clinical challenge in the treatment of myocardial infarction, and currently, there is a lack of effective therapies. Chen *et al.* [253] constructed a dual system of an MOF intelligent carrier, which utilized its pH-responsive property to enable the escape of endosomes and avoid lysosomal degradation. The dual system, composed of the intelligent carrier and conductive micro-needle patches, achieved targeted, long-lasting efficacy and multifunctionality. This provides a new synergistic strategy that combines gene regulation and electrical integration for I/RI treatment (Figure 16).

In the field of SDVGs, while suppressing pathological responses, achieving rapid and healthy endothelialization remains a key challenge in cardiovascular implant therapy. Zheng *et al.* [254] proposed a strategy of ultrasonic-driven directional arrangement of piezoelectric nanomaterials, which utilizes exogenous ultrasonic energy to enhance the electrical stimulation performance of endogenous piezoelectric fibers and stimulate the release of anticoagulant drugs within the fibers. This innovative approach confers upon cardiovascular implant devices bespoke surface characteristics that afford exquisite spatiotemporal control over interface/tissue responses, thereby orchestrating the functional endothelialization cascade and culminating in the regeneration of an optimal neointima. The vascular stent used in this study demonstrated the comprehensive functionality and clinical relevance of this advanced technique.

### Neural injury repair

Neural injuries and diseases are the main causes of functional impairment globally, and effective treatment methods are urgently needed. Neurological recovery and restorative therapies are considered the most promising approaches for restoring neural functions [17]. The goal of neuroengineering is to create tools for investigating, restoring and augmenting the functions of the nervous system, to tackle problems linked to nerve damage and neurological disorders, and to improve brain function. The main obstacles in the development of neural engineering include insufficient understanding of the properties of brain tissues, lack of theoretical support for the interactions of biological macromolecules, insufficient research methods for studying dynamic activities within neural tissues, unclear mechanisms of neural connectivity and limited treatment options for neurological disorders [17]. The synergistic integration of intelligent biomaterials within the framework of neuroengineering engenders substantial optimism for surmounting the formidable challenges intrinsic to brain science. Successful neural regeneration requires materials to provide a dynamic platform for multifunctional integration. This will empower us to better simulate and study the neural environment and to design new therapeutic strategies, paving the way for substantial progress in the treatment of neurological diseases and the improvement of neural health.

Traditional neural implant devices do not match the mechanical properties (such as flexibility) of biological tissues, which can easily lead to foreign body reactions [255]. The primary objective of bionic electronics is to accurately replicate the mechanical properties of biological tissues. By employing soft and flexible designs, as well as natural or artificial substances such as polymeric and hydrogel-based materials, stiffness can be reduced to achieve better mechanical compatibility with biological tissues, thereby enhancing integration and minimizing tissue trauma [235].

**Figure 17:**
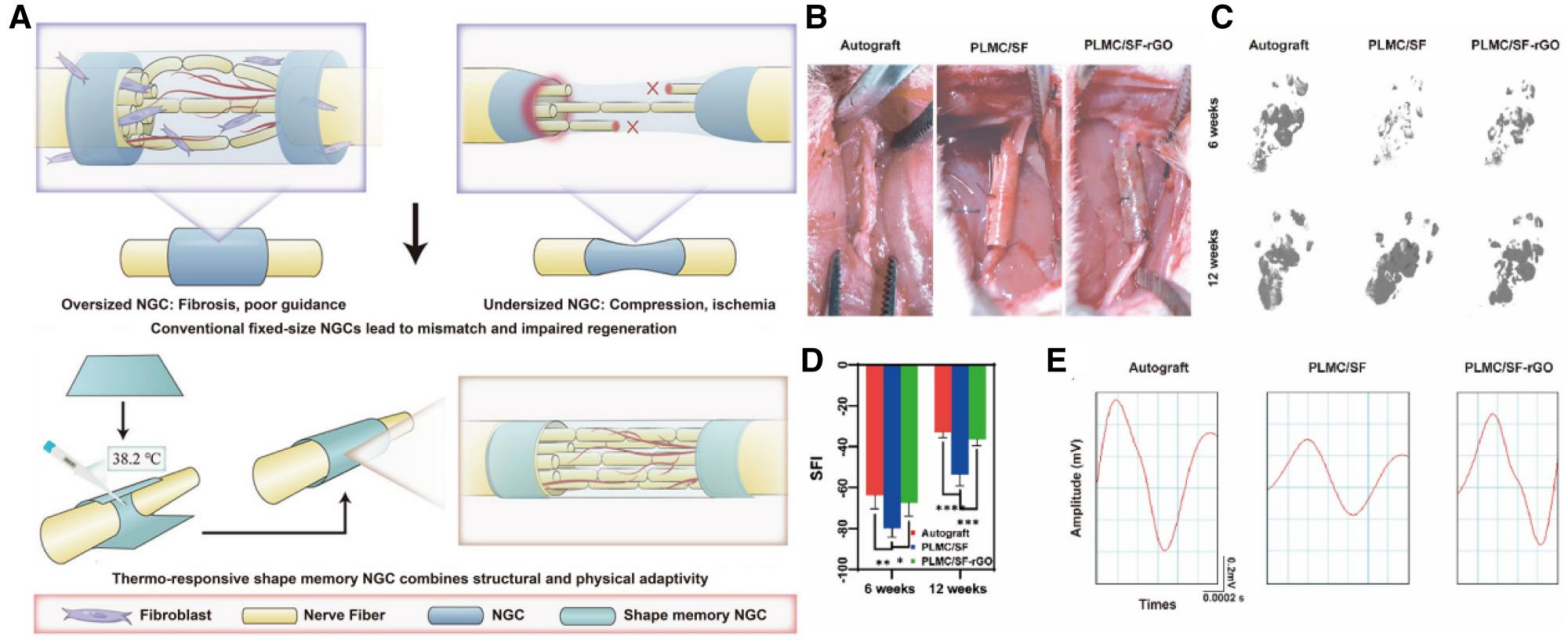
Intelligent biomimetic materials for the repair of nerve injuries. (**A**) Excessive nerve guiding tubes will reduce the guiding efficiency and promote the proliferation of fibrous tissues, while too small nerve guiding tubes will cause distortion and ischemic compression. Nerve guiding tubes with thermally responsive shape memory properties can autonomously coil up, thereby fully enveloping the nerve, achieving tension-free anastomosis and achieving the best anatomical alignment effect. (**B**) Images depicting autograft and nerve guidance conduit implantation. (**C**) Rat footprint map at 6 and 12 weeks. (**D**) Sciatic functional indices at 6 and 12 weeks. (*n* = 5). (E) Electromyography signals obtained at 12 weeks. Reproduced with permission from Ref. [256]. Copyright 2025 © Elsevier.

Boufidis *et al.* [235] successfully reduced the mechanical performance mismatch and inflammatory response at the electrode-tissue interface by using flexible polymers and hydrogels, thereby significantly enhancing the biointegration and long-term stability of the device. Patients with neural injury, a severe form of neurological damage, often suffer from lower limb and urinary dysfunction, which dramatically diminishes their quality of life. Gao *et al.* [256] designed a thermally responsive self-curving multichannel nerve guiding catheter, which is composed of shape memory poly (lactide-co-trimethylene carbonate) (PLMC), silk fibroin (SF) and reduced graphene oxide (rGO). It can achieve scarless regeneration and match the individualized anatomical structure. This electrospun PLMC/SF-rGO catheter has axially arranged fibers inside, which can provide topological guiding signals to achieve directional guidance, and has programmable shape recovery ability. It can be adjusted individually during the operation and maintain shape stability throughout the nerve regeneration process, providing a high-potential autologous graft alternative for scarless personalized nerve repair (Figure 17).

### Precise drug delivery and cancer treatment

Precise drug release is a key objective of drug delivery systems, aiming to efficiently and specifically deliver drugs to the lesion site while minimizing toxicity to normal tissues [257]. Common approaches for precise release include using materials such as nanoparticles, liposomes and polymer carriers, and achieving drug release under specific conditions through mechanisms such as targeting ligands, pH sensitivity and temperature sensitivity [258–260]. Additionally, molecular machines in drug delivery systems mainly achieve programmable drug loading and on-demand release through mechanically interlocked molecules. The core molecular framework includes rotaxanes, chain-like hydrocarbons, mechanical force-responsive groups and so on [261]. Sheng *et al.* [257] systematically adjusted the drug release by modifying the structures of the stopper and the crown ether macrocycle, thereby regulating the speed and schedule of drug release. This research offers fresh ideas toward the advancement of novel molecular machines and drug delivery platforms. We can anticipate the application of this molecular-machine-based drug release system in the treatment of more diseases, safeguarding human health.

**Figure 18:**
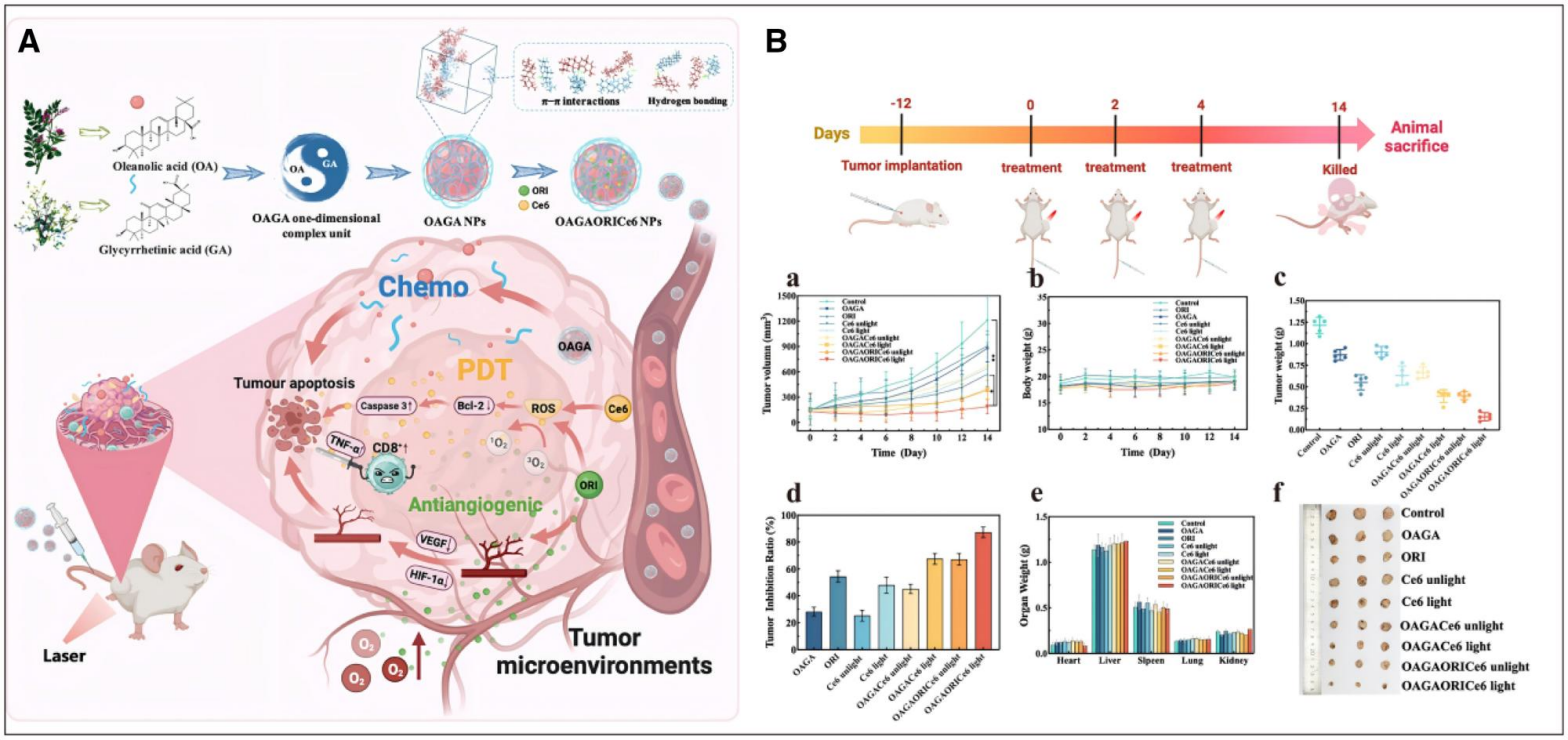
Representative cases of precise drug delivery. (**A**) The synthesis process of the OAGAORICe6 nano-assemblies with chemical, photodynamic therapy and anti-angiogenic effects. (**B**) Diagram of the study timeline in 4T1 tumor‑bearing mice: (a) tumor volume alterations over 14 days in 4T1 mice. (b) Body weight fluctuations in 4T1 mice over 14 days. (c) Excised tumor mass following intravenous injection in mice. (d) Tumor growth inhibition rate of mice across different treatment groups after 14 days (*P < 0.05 and **P < 0.01). (e) Mass of major organs harvested from mice following intravenous injection. (f) Images of tumor tissue slices from each treatment cohort after 14 days. Data are mean ± SD (*n* ≥ 3), and the n represents biologically independent experiments. (*P < 0.05, **P < 0.01 and ns represented nonsignificance). Reproduced with permission from Ref. [268]. Copyright 2024 © Wiley-VCH GmbH.

Compared with traditional biomaterials, nanostructures have significant advantages in specific surface area, electrochemical activity, cell interactions and guiding cell behavior [262, 263]. Nanostructures exhibit considerable promise for use in the domain of smart biomaterial design. For instance, nanomaterials have been designed to target bone metastases, enabling combined tumor therapy and bone regeneration [264]. Shape-convertible nanomedicines are a type of intelligent drug delivery system that can change their size, shape, surface charge or surface chemistry under specific stimuli, aiming to overcome the conflicting requirements of different physiological barriers for nanomedicines [265, 266]. Specific material systems include polymer micelles modified with enzyme-responsive peptide segments, polymer vesicles with pH-responsive cinchonidine bonds, block copolymers with photoreactive azobenzene groups and so on [267]. Wang *et al.* [268] researched and assembled the shape-convertible nanomedicines. The nanoparticles transformed into a nanofiber form in the tumor microenvironment, enabling targeted drug delivery for synergistic enhancement of photodynamic therapy. The smart responsive approach used in this study demonstrates considerable potential for advancing the development of innovative combinatorial treatments for cancer and various other diseases (Figure 18).

The concept of programmable matter was first introduced by Toffoli and Margolus in 1991 [269]. Programmable materials are advanced substances designed to undergo precise and controlled changes in their morphology, physical properties and functional behaviors in accordance with predefined stimuli or external conditions [269]. Programmable materials can change their morphology and physical performance characteristics as needed, attracting extensive attention. DNA presents unique advantages to enable accurate control of drug delivery. The sequence-specific programmability of DNA allows for the design of functional structures capable of intelligent molecular recognition and targeted release, providing a powerful platform for controlled delivery systems [270]. Yerneni *et al.* [271] ingeniously leveraged the functional and programmable properties of DNA to design a cholesterol-modified DNA strand and introduced a nitrophenyl group as a photocleavable linker, enabling the exosomes to be released upon light stimulation and extending the release time.

The nano-drug delivery platform has gradually emerged as a research focus in the realm of tumor treatment in recent years. Traditional anticancer drugs face challenges such as high toxicity and uneven efficacy, which limit their application scope and usage frequency. The Korean PharmaResearch company’s PRD-101, based on the DOT nanoparticle platform, has received FDA IND approval and will launch the first human Phase I clinical trial. Different from traditional anticancer drugs, the delivery system of PRD-101 is based on optimized DNA fragments, which are generated through the company’s proprietary DOT platform technology. The core advantage of this platform resides in its capacity to accurately regulate the immunogenicity, pharmacokinetic properties and biodistribution of the drug, improving the drug loading efficiency and enhancing its therapeutic effect, providing patients with a better treatment experience.

The currently available intelligent delivery systems include Doxil®, Abraxane® and Onivyde® [272–274]. Doxil® modifies the surface of liposomes with hydrophilic polymer polyethylene glycol (PEG) to form a hydration layer, evading recognition by the mononuclear phagocytic system, significantly reducing cardiac toxicity and prolonging circulation time [275, 276]. This demonstrates that long-circulating nanocarriers can improve drug safety. It is the pioneer in the clinical transformation of nanomedicines [277]. Abraxane® uses human serum albumin as a carrier and combines hydrophobic paclitaxel to form 130-nanometer particles. It has been demonstrated that using endogenous proteins as carriers can solve the problem of drug solubility and enhance the therapeutic index [273]. This is a successful example of the biomimetic strategy. Onivyde® adopts a dual-chamber liposome structure, encapsulating irinotecan and its active metabolite SN-38 in a stable form [275, 278]. This has demonstrated that in highly aggressive cancers with limited treatment options, nanomedicines can provide clear survival benefits [279, 280]. This lays the groundwork for more intelligent material design in the years ahead. The future precise drug delivery systems should focus more on advanced functions such as active targeting, controlled release and immune regulation [281].

### Personalized implants and organ chips

Personalized medicine fundamentally relies on the ability to customize treatment intervention measures based on individual patient conditions. The design of intelligent biomimetic biomaterials provides a crucial supporting platform for this. For example, materials that are sensitive to pH values, temperatures or enzyme signals enable them to interact intelligently with biological signals, thereby transforming inert scaffolds into platforms with autonomous immune regulation capabilities. Personalized medicine offers biological inputs tailored to individual patients, including information from genomic data, disease phenotypes and anatomical imaging. These pieces of information directly guide the engineering design of responsive materials [282].

**Figure 19:**
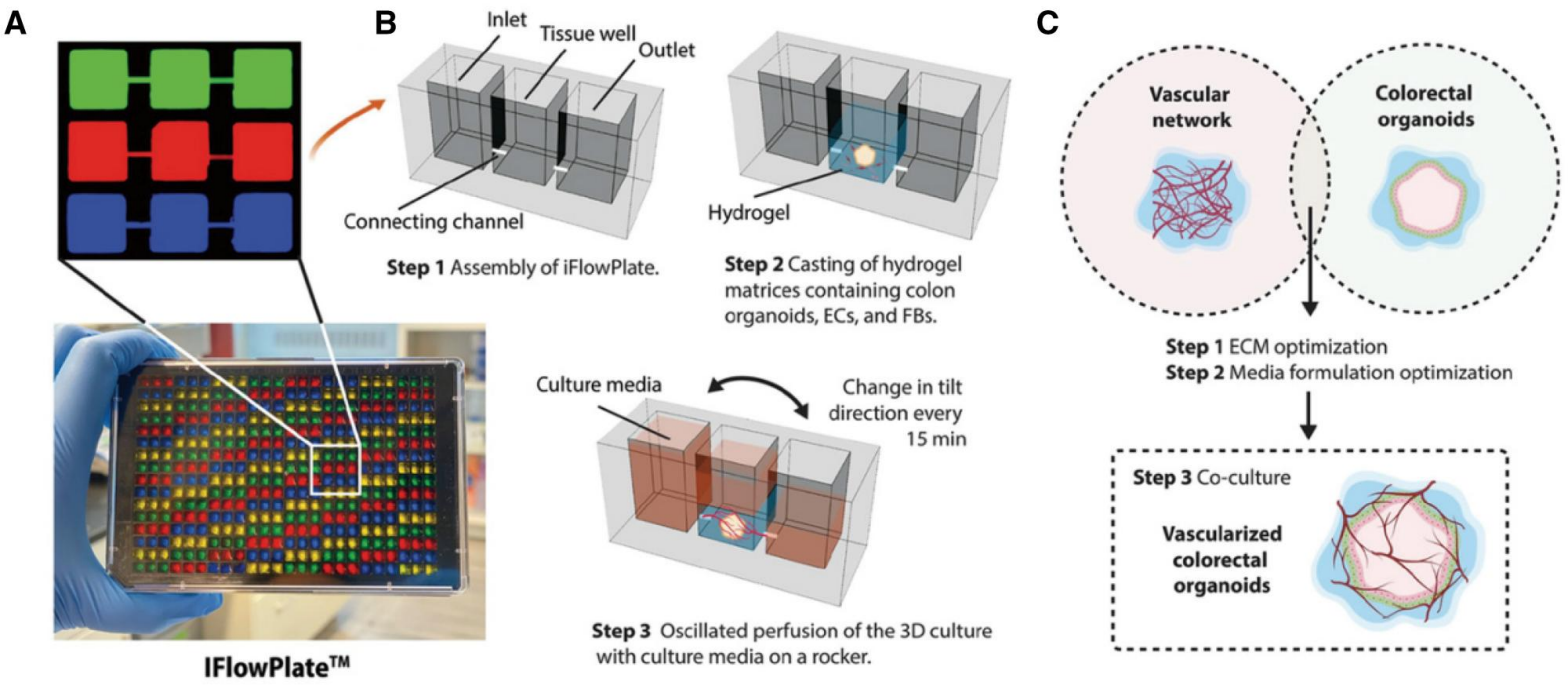
Structure and operation steps of the organ chip. (**A**) Diagram showing 128 independent reaction units of the organ chip distinguished by different dyes. (**B, C**) Schematic diagrams of the microfluidic channels and vascularized intestinal organ cultures in a single reaction unit. Reproduced with permission from Ref. [286]. Copyright 2020 © Wiley-VCH GmbH.

The realization of personalized implants relies fundamentally on 3D/4D printing technology [283]. It can not only precisely replicate the complex craniofacial bone defect shapes based on CT data but also manufacture intelligent structures with mechanical gradients or stimulus-responsive capabilities through multimaterial printing [135]. For instance, the 4D-printed shape-memory polymer scaffold can undergo preset deformation at body temperature, achieving dynamic fitting after minimally invasive implantation [126]. Organ-on-a-chip technology provides a transformative platform for this purpose. By culturing patient-derived cells in microfluidic chips and recreating the tissue microenvironment (such as blood flow shear stress and tissue interfaces), highly predictive individualized disease models can be constructed [284]. A heart chip that integrates patient-derived cardiomyocytes, endothelial cells and immune cells can assess the risk of inflammation or fibrosis caused by implanted materials [285]. Rajasekar *et al.* [286] designed an organ chip that can be used *in vitro* and can independently cultivate up to 128 perfusable vascularized organoid models at one time. This chip has open reaction units and operates without an external pump system, and additionally, the cultivated models are convenient for tissue extraction for subsequent histological or transplantation purposes. Through the optimal preparation of extracellular matrix and culture medium, patient-derived intestinal organoids can successfully co-culture with the self-assembled vascular network, promoting the growth of organoids. Using this organ chip can unlock more opportunities to identify potential therapeutic targets or develop disease-related models (Figure 19).

In the future, AI will accelerate this process, forming a rapid iterative closed loop of design-chip verification-manufacturing. System-level verification will be conducted on multi-organ chips [287]. Although there are challenges such as standardization, cost and regulatory approval, this path undoubtedly will bring the clinical transformation of biomaterials into a truly precise and efficient new era.

## Challenge and future prospects

In the context of advancing science and technology, intelligent and bionic biomaterials have made impressive progress in medicine. However, there are still great challenges for translating the laboratory findings to clinical applications [17, 288, 289]. We conducted a detailed discussion on the following aspects: challenges in the material-biological interface, integration challenges of advanced technologies (such as AI, 4D printing and synthetic biology), clinical applications and regulatory challenges.

### Material-biological interface interaction

A deep comprehension of the processes governing cell-biomaterial interactions facilitates the definition of design criteria for biomaterials. The current unclear mechanism results from a lack of cross-scale, real-time and dynamic understanding of the complex process of material-biological interface interactions. Future research should combine cross-disciplinary strategies, encompassing materials science, immunology, cell biology and mechanics [290]. It is very necessary to develop cross-scale *in situ* characterization technologies (such as live imaging and micro-region omics) to analyze in real time the evolution of material surfaces, the dynamic behavior of immune cells and intercellular communication.

After being implanted into the human body, the host’s immune system will trigger a series of complex biological reactions. Although the functional design of intelligent and biomimetic biomaterials has made significant progress, solving the general problems at the material-biological interface requires addressing specific issues such as immune responses, fibrosis and long-term integration [153]. Biomaterials modulate the immunological response via their physical and chemical properties and the release of temporally controlled immunomodulatory factors. When macrophages polarize to the pro-regenerative M2 phenotype, it is conducive to tissue repair and material integration. Meanwhile, fibrosis is a major clinical problem at the interface between materials and biology. Fibrosis directly hinders the functional integration of implants with the host and can also trigger secondary complications such as infections. Therefore, antifibrosis is the core bottleneck that must be overcome to achieve long-term integration [291, 292]. Additionally, it is of great significance to establish a more systematic long-term integrated assessment standard, which should cover multiple dimensions such as short-term biocompatibility, long-term tissue functionality and cross-individual differences.

Meanwhile, the concept of bio-adaptive materials emerged. Tissue repair is a highly dynamic and phased process, from hemostasis and inflammation to cell proliferation and tissue remodeling. Each stage has distinct requirements for the microenvironment and single-function responsive materials are difficult to meet the time-controlled requirements throughout the entire repair cycle [293, 294]. Bioadaptive materials refer to those that, during the process of tissue repair, can autonomously adjust their functions over time in response to the dynamic changes at different stages of repair—from the antibacterial and anti-inflammatory functions in the early stage to the promotion of angiogenesis, cell proliferation and tissue regeneration in the later stages, establishing a two-way adaptive and co-evolutionary relationship between the material functions and the tissue repair process [295–297]. Cui *et al.* [298] endowed inert metal implant devices with ‘self-renewal’ surface properties, enabling them to possess the biological functions of releasing antibacterial, anti-inflammatory substances and promoting tissue repair on demand in a gradual manner.

The researchers drew inspiration from the multilayered interlocking structure of the shells of soft-bodied animals, the adhesion chemical mechanism of mussels and the self-renewal active mucus layer of corals and constructed an *in situ* sandwich-like functional interface. This functional interface is composed of a microstructured prelayer derived from the substrate, a mussel-inspired bioadhesive interlayer and a therapeutic active dynamic hydrogel top layer. The dynamic bonds, such as borate ester bonds and imine bonds in the hydrogel, enable it to respond to pathological signals such as acidic pH and ROS, locally releasing antibacterial and anti-inflammatory components efficiently, while exposing the microstructured prelayer that is conducive to cell growth at the right time.

Future research should focus on specific clinical application scenarios, clearly identify the core contradictions of long-term integration, prioritize the material design based on these contradictions, and explore more integrated diagnostic and therapeutic biomaterials.

### Integration of multiple advanced technologies

The integration of various advanced technologies (such as AI, 4D printing and synthetic biology) faces significant feasibility bottlenecks and cost barriers [299, 300]. Although the application of AI in the design of biological materials has made remarkable progress, it has long been hindered by the lack of high-quality labeled data [301]. Materials informatics and chemical informatics have always faced the challenge of data scarcity. This seriously hinders the extraction of the important relationship between structure and performance [302]. To tackle this problem, the research team developed a series of approaches, such as self-supervised learning of unlabeled structural data, transfer learning from related fields, active learning for intelligent sample selection and integrating basic physical laws as inductive biases into physically oriented neural networks [303].

Model interpretability is also a crucial challenge, especially in the field of biomedicine, as safety and regulatory acceptance require a mechanistic understanding [304]. Technologies including Shapley Additive exPlanations and Local Interpretable Model-agnostic Explanations, which are interpretable AI techniques, have been increasingly adopted to rank feature importance and explain model predictions [305]. However, the balance between prediction precision and explainability remains an area that requires further investigation. The limitations of 4D printing remain, including the shortage of environmentally benign and mechanically strong bio-inks, the energy-demanding manufacturing process, elevated manufacturing expenses, difficulties in scaling up, reproducibility problems and regulatory obstacles that impede clinical translation [306]. For instance, the strategy of reducing the usage of SMPs by physically blending or layering them with conventional polymers to lower costs is expected to address some economic issues.

However, how to maintain mechanical properties and shape recovery behavior while achieving cost reduction remains an open engineering problem [307]. Engineered Living Materials (ELMs) are created by merging synthetic biology and materials engineering, resulting in dynamic response systems possessing living functionalities. Nevertheless, the medical application of ELMs faces multiple difficulties, including biological safety, scalability and regulatory issues [308]. The integration cost of advanced technologies is much greater than the cost of conventional biological materials, and the scarcity of interdisciplinary talents further increases the labor costs [306, 309].

### Regulatory challenges

The intelligent and bionic materials pose great challenges to the existing regulatory framework for medical devices/drug-device combination products, requiring new evaluation standards and review pathways. In terms of standardization, the dynamic degradation behavior, stimulus-responsive characteristics and long-term performance changes after implantation of intelligent biomaterials, among other key attributes, have not been fully covered by the existing standards [310].

Currently, the standards mainly focus on static absorbable materials rather than intelligent systems with dynamic response capabilities [311]. The existing regulatory framework still uses traditional methods for the assessment of drugs and biological products, resulting in reviews that typically rely on case-by-case judgments and a lack of a unified standard [311].

Moreover, there are differences in the interpretation and implementation standards of the same international standard in different jurisdictions, which increases the compliance costs for global submissions. Intelligent and bionic biomaterials, due to their multiple attributes of being drugs, devices and biological products, encounter unique challenges in classification and path selection during regulatory review. Although regulatory frameworks in different jurisdictions have their own focuses, they share a convergent core logic, with the primary mode of action (PMOA) of the product serving as the fundamental criterion for determining the review category. At the same time, combined with risk stratification and the collaborative review mechanism for combined products, the safety and efficacy of complex products are ensured to be scientifically evaluated.

The dynamic, adaptive and even evolutionary nature of intelligent/bionic materials fundamentally conflicts with the traditional regulatory assumption of being static, uniform and preset. Future regulatory science must undergo fundamental innovations in product definition, evaluation tools and review processes. The regulatory framework must “co-evolve” with material technology. Regulation is not just about passively setting thresholds; it should actively shape an ecosystem that ensures safety while promoting innovation.

## Conclusions

At present, biomaterials have undergone a fundamental transition from serving as passive structural supports to functioning as active regulators of physiological processes by integrating intelligent response mechanisms and biomimetic functionality in the context of advanced technologies, like 4D printing and AI-assisted processes. They demonstrate the potential to transform clinical treatment approaches in areas including difficult-to-heal wound repair, complex tissue regeneration and precision drug delivery.

The review provided readers with a comprehensive framework for intelligent responsive materials and biomimetic functional materials: 4D printing, precise molding technologies, AI-based material design platforms and *in vivo* monitoring technologies. Six main progress areas and clinical applications are introduced: skin wound repair, bone and cartilage regeneration, cardiovascular diseases, nerve injury repair, precise drug delivery and cancer treatment, as well as personalized implants and organ chips. The existing obstacles and prospects of intelligent biomimetic biological materials are also discussed in detail. Further research should concentrate on the transition from “responsive” materials to “life-adaptive” materials with bidirectional interaction capabilities, enabling them to autonomously regulate functions according to different stages of tissue repair. In terms of clinical translation, these materials have significant application potential in areas including precise targeted drug delivery, infectious bone defect repair and intelligent cardiovascular stents. However, the core bottleneck for truly transitioning smart biomimetic materials from the laboratory to clinical application lies in deciphering the dynamic interaction mechanisms at the cross-scale “material-biological” interface. Additionally, the long-term functional stability and immunocompatibility of these materials following prolonged implantation still require validation.

Future breakthroughs will depend not only on deep integration of multidisciplinary technologies to accelerate design iteration but also on establishing novel regulatory evaluation systems tailored for such dynamically evolving materials. Furthermore, the traditional medical device approval framework is based on static products, while intelligent biomimetic materials have the characteristic of dynamic changes (in the time dimension). Therefore, it is strongly recommended that researchers closely collaborate with regulatory agencies to establish a new standardized testing protocol and regulatory approval framework that is adapted to dynamically evolving materials, thus truly achieving the transition from the laboratory to clinical applications. This will ultimately enable profound functional and structural symbiosis between artificial materials and living organisms.
